# Earth's Hadean crust formed via operation of convergent tectonics

**DOI:** 10.1093/nsr/nwaf230

**Published:** 2025-06-02

**Authors:** Denggang Lu, Jia Liu, Qunke Xia, Zhikang Luan, Jingjun Zhou, Tianting Lei, Lu Wang, Eero Hanski

**Affiliations:** Research Center for Earth and Planetary Material Sciences, School of Earth Sciences, Zhejiang University, Hangzhou 310058, China; Research Center for Earth and Planetary Material Sciences, School of Earth Sciences, Zhejiang University, Hangzhou 310058, China; Research Center for Earth and Planetary Material Sciences, School of Earth Sciences, Zhejiang University, Hangzhou 310058, China; Research Center for Earth and Planetary Material Sciences, School of Earth Sciences, Zhejiang University, Hangzhou 310058, China; Research Center for Earth and Planetary Material Sciences, School of Earth Sciences, Zhejiang University, Hangzhou 310058, China; Research Center for Earth and Planetary Material Sciences, School of Earth Sciences, Zhejiang University, Hangzhou 310058, China; Key Laboratory of Silicate Cultural Relics Conservation, School of Cultural Heritage and Information Management, Shanghai University, Shanghai 200444, China; Oulu Mining School, University of Oulu, Oulu 90014, Finland

**Keywords:** Hadean crust, zircon, TTG, potassic granites, convergent plate

## Abstract

Determining the composition, formation mechanisms and stability of the Hadean continental crust is essential for understanding the early geological history of Earth. Detrital zircons, largely from Jack Hills of Western Australia, provide the dominant direct records for the nature of continental crust during the Hadean eon and its formation processes. Although isotope and trace element compositions of these zircons are extensively determined, the major and trace element compositions of their host rocks and corresponding parental magmas remain largely debated, making the nature and evolution of the early Earth's crust ambiguous. Here, based on the comprehensive datasets for global magmatic zircons and their host rocks, we have developed machine learning models to reconstruct multiple major and trace element concentrations of the parental magmas from which Jack Hills zircons grew. The results show that the Hadean continental crust had SiO₂ contents ranging from 58 to 78 wt% with K₂O/Na₂O and Sr/Y ratios in the range of 0.1–1.2, and 1–103, respectively. It was generally not andesitic in lithochemistry, but rather felsic and dominated by low- to medium-pressure tonalite–trondhjemite–granodiorite (TTG) and potassic granites. These rocks would be derived from partial melting of low- and high-potassium mafic proto-crust, respectively, with the latter also incorporating contributions from tonalite. The lack of high-pressure TTGs does not preclude their formation at convergent plate margins, but suggests that the Hadean felsic crust would originate from the collisionally thickened rather than the deeply subducted oceanic crust. Therefore, the formation of continental crust on the Hadean Earth can be explained by the operation of convergent tectonics, outlining a petrogenetic model for Archean TTG rocks.

## Teaser

Machine learning models were built to predict the bulk rock compositions based on the trace elemental pattern of zircons. The applications of these models to the Jack Hills zircons demonstrate Earth's Hadean crust formed via operation of convergent tectonics.

## INTRODUCTION

Understanding the composition of the Earth's earliest continental crust and constraining its tectonic setting are crucial to deciphering the planet's formative and differentiation processes. In the absence of rock records older than 4.02 Ga, the only direct clues to the Hadean Earth come from rare detrital zircons, predominantly found in metasediments of Jack Hills in Western Australia and the Green Sandstone Bed in the Barberton greenstone belt in South Africa [1–3]. While it is generally agreed that these Jack Hills zircons (JHZs) were derived from erosion of igneous rocks, the nature of their parental magmas and the associated geodynamic regime remain controversial [2,4–10].

Most of the previous studies have focused on zircon U–Pb ages and Hf or O isotope compositions, providing information not only on the time since the crustal source differentiated from the primitive mantle, but also on whether the source contained components that interacted with surface water under different conditions, as well as the origin of the Hadean crust in subduction or non-subduction settings [6,8,11–19]. Based on selected trace element ratios of zircons and traditional discrimination diagrams (e.g. Th/Y, Th/Nb), the parental magmas of JHZs have been suggested to be either andesitic, formed in a modern-like arc setting [8,20], or granitic, resulting from partial melting of mantle-derived mafic rocks in a non-subduction setting [3]. Furthermore, machine learning (ML) models trained exclusively on zircon data from specific granite types (I- or S-type) or tonalite–trondhjemite–granodiorite (TTG) rocks have classified the JHZ parental magmas accordingly, though this approach inherently excludes other potential rock types [21–23].

Different opinions have largely been caused by the long-term absence of reliable approaches to quantitatively recover the compositions of host rocks in terms of multiple major and trace elements, including the covariations of major and trace elements, which are indicative of the nature of host rocks, the source characteristics and the forming conditions. For instance, K_2_O contents and K_2_O/Na_2_O ratios at given SiO_2_ contents are critical for identifying TTGs from sodium or potassic granites [24,25], and Sr/Y ratios are more indicative than heavy rare earth element (HREE) extended fractionation for inferring the depth of either partial melting or fractional crystallization [26,27]. However, the major elements like K_2_O and Na_2_O are difficult to acquire through the reverse calculations from zircon composition, due to the lack of precise analysis of these elements in zircons and the corresponding accurate partitioning coefficients [20,21].

Numerous studies have revealed that in many natural samples the trace elemental features of igneous zircons are closely linked to the composition of their hosting rocks in both levels of major and trace elements, which were attributed to the source heterogeneity or magmatic differentiation [28–30]. ML regression models can handle a larger number of features and capture complex, non-linear relationships within extensive datasets [31,32], which provides us with a powerful tool to explore the internal correlations between the composition of igneous zircons and the parental magma in which they crystallized. In this work, we developed new ML models that provide quantitative, reliable and self-consistent constraints on multiple element concentrations of the Earth's early crust, with the advantages of no need to assume the parental magma type and partitioning coefficient as prerequisites. We have compiled the most comprehensive datasets of global magmatic zircon compositions and their host rocks so far. By utilizing these datasets, we employed supervised ML algorithms to develop models that predict the major and trace element compositions of host rocks from which the zircons would crystallize, provided that the zircon's element composition is available from known data (for more details, see Supplementary Data). These models learn directly from data without relying on predefined functional forms, improving prediction accuracy and revealing subtle patterns and variable interactions that may be challenging to detect through conventional approaches. By using ML, we have reconstructed the compositions of the parental magma for the JHZs and tracked their magma genesis. Based on these data, we discuss the rock types of continental crust on early Earth, partial melting depth and potential geodynamic implications.

## DATA AND METHODS

We have compiled geochemical data for 14 241 magmatic zircons and their host rocks (*n* = 823) covering a large range of geochemical compositions (from mafic to felsic in lithochemistry, and from enriched to depleted in geochemistry), formation ages (from the Archean through the Proterozoic to the Phanerozoic) and geodynamic settings (e.g. accretionary, collisional and intracontinental orogens) (detailed information can be found in Figs S1 and S2 and Supplementary material). During data collection, we checked cathodoluminescence (CL) images and analytical methods [e.g. small-spot secondary ion mass spectrometry (SIMS)] from accessible original literature to ensure that the trace element data and U–Pb dating were obtained from the same or closely related structural domains. These zircon data were filtered according to the following criteria to ensure the magmatic origin of the zircons [33–35]: (i) light rare earth element index (LREE-I) ≥ 30; (ii) La ≤ 0.5 ppm; (iii) Ca ≤ 150 ppm, Fe ≤ 150 ppm (if available); and (iv) Th/U ≥ 0.1. These filtering rules can also exclude the potential effects from the mineral inclusions in zircons. After excluding the zircons affected by later alterations, we have obtained data for 9879 zircons and 695 host rocks.

We randomly split the dataset into training data (90% of the data) and testing data (10% of the data). The relationships among element content of whole-rock (output data), selected trace element compositions of zircons and crystallization age (input parameters) were trained through several ML algorithms (e.g. Extreme Gradient Boosting, XGBoost), artificial neural networks and random forest) in the training dataset. The models were then applied to the testing dataset, which was never used during the training process, and the predicted element contents of host rocks were compared with the referenced values to evaluate the model's performance based on two metrics: R^2^ (determination coefficient) and root mean squared error (RMSE) (Supplementary Data). We have also conducted 10-fold cross-validations to minimize overfitting and more accurately estimate the model's generalization ability on unknown data (Supplementary Data), and found that the XGBoost algorithm can result in the best performances (Table S1).

**Figure 1.**
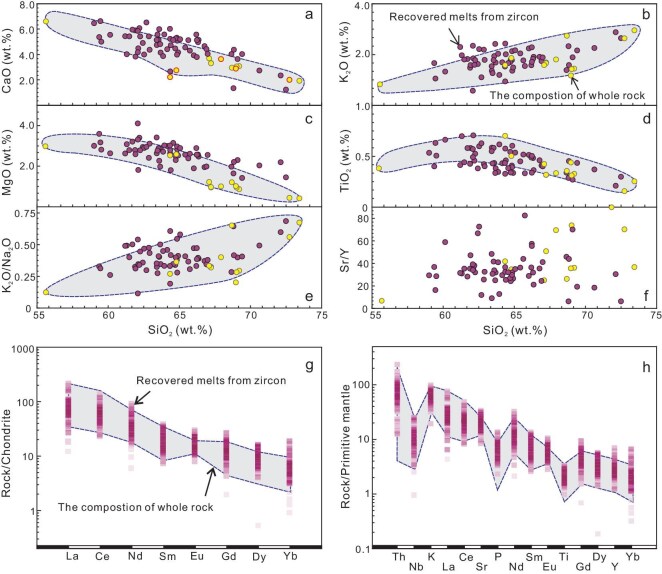
Testing the ML model to recover major and trace element concentrations of magma from zircon. (a–f) Key major element relationships of melt calculated from zircon of the Tarim case [34]. (g) Chondrite-normalized rare earth element diagram, and (h) primitive mantle-normalized trace element diagram for melts calculated from zircon compositions (normalization values from Sun and McDonough [55]). Red hollow circles represent samples with trace element analyses of zircons, and the color gradient in Fig. 1g and h represents sample distribution density.

Using the XGBoost algorithm, the R^2^ values from 10-fold cross-validation range from 0.68 to 0.85 (mostly between 0.75 and 0.85), and show only minor variation across individual elements, indicating that the model performs robustly without signs of overfitting. In addition, the R^2^ scores of the testing data are from 0.75 to 0.84, comparable with the R^2^ values in several recent ML regression studies [36,37], indicating good regression performances (Figs S3 and S4 and Table S2). Here, we estimate the uncertainty of the predicted results (e.g. for SiO_2_, RMSE = ±3.8 wt%, see Table S2 and Figs S3–S5 for others) with the calculated RMSE from the predicted results for the testing data, which results in typical relative uncertainty at the level of ±20% (Table S2). We have further applied the predicting models to the magmatic zircons for several suites of rocks, with ages from Cenozoic orogens to ∼4.0 Ga Acasta Gneiss samples (Fig. S6), which were not used for training the models. The results show that for each case, the recovered concentrations of major and trace elements, and the resulting primitive mantle-normalized trace element patterns, Sr/Y ratios and the identified category in the source lithology discrimination diagram are consistent with the records of the rocks (Fig. 1, Figs S6–S8). What's more, the recovered co-variations among different major and trace elements (e.g. SiO_2_ vs. other oxidized and trace elements) are in line with those demonstrated by these rock suites (Fig. 1, Figs S6–S8).

## RESULTS AND DISCUSSION

### Compositions of the parental magmas for JHZs recovered by the ML model

Following the same geochemical screening as used for the training data [33–35] (see Supplementary  Data, for details), we compiled 488 igneous JHZs, with concordant U–Pb ages (from 4364 to 3300 Ma) and trace element concentration data (Supplementary material). For the collection of the JHZ dataset, we examined the CL images and analytical methods (e.g. small-spot SIMS) reported in the original literature to ensure that the trace elements and U–Pb dating were obtained from the same or closely related structural domains while identifying potential mineral inclusions and fractures. Few zircons in this study have combined trace element, Hf and O isotope analyses. Both the element concentrations and patterns are within the range of the data used for training the ML model (Figs S3 and S4). Based on the trace element data of these zircons and the ML models, the major and trace element compositions of parental magma for JHZs were recovered (Supplementary material, Fig. 2 and Fig. S9). It should be emphasized that while the parental magma of zircons may differ from fully crystallized rocks (such as granitoids), the application of our models to various cases (e.g. 3.2 Ga granitoids in Tarim) demonstrates that the compositional range of the recovered parental magmas shows remarkable consistency with that of the bulk rocks (Fig. 1). In the subsequent discussion, the term ‘parental magma’ is employed to denote the igneous rock sources from which the JHZs were derived.

**Figure 2.**
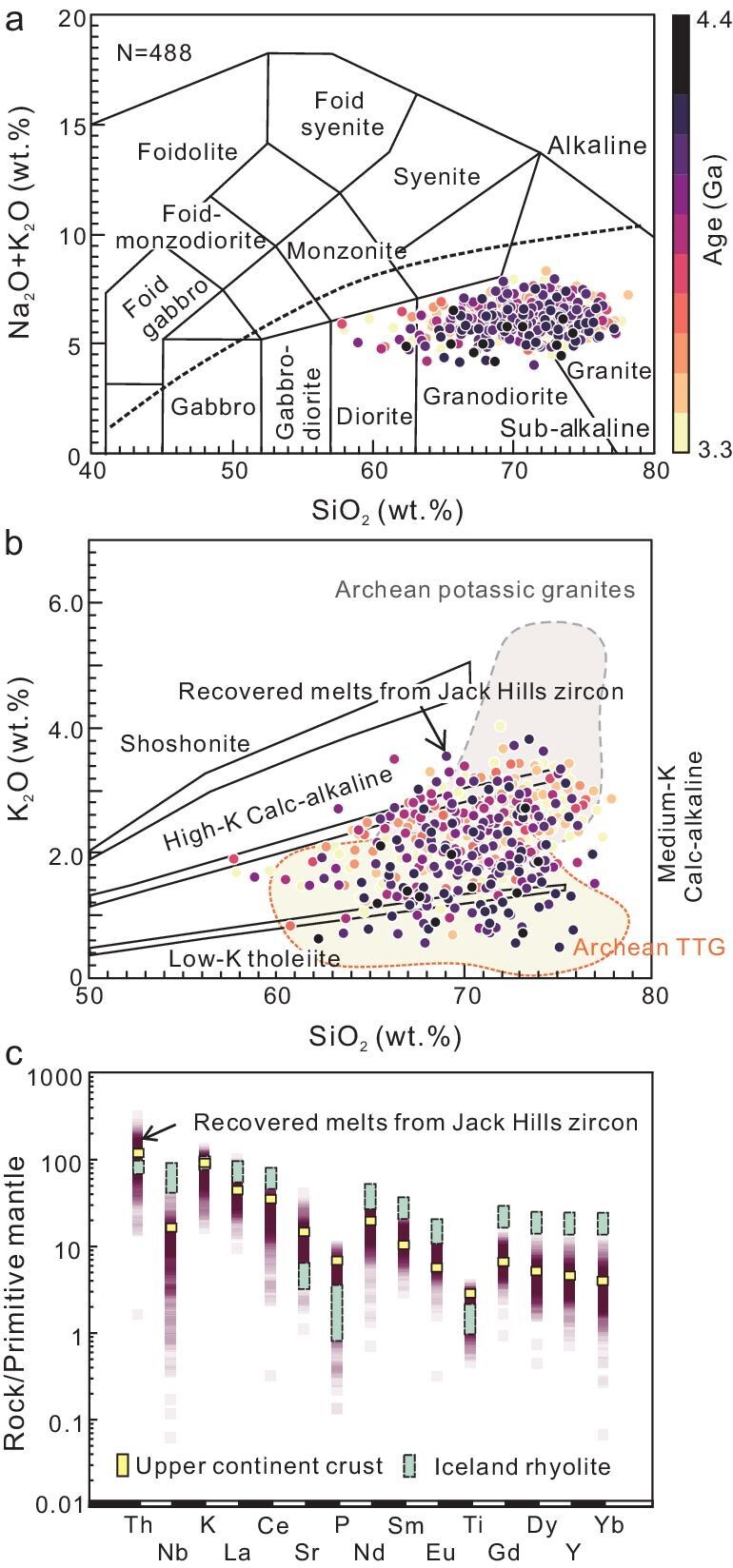
Geochemical diagrams of parental magma of Jack Hills detrital zircons. (a) Total alkalis vs. silica (TAS) diagram [56]. The color gradient in the figure represents the sample ages. (b) K_2_O vs. SiO_2_ diagram [57] (fields for Archean TTG and potassic granites are based on Moyen [27]); and (c) primitive mantle-normalized [55] trace element diagram for parent magma of Jack Hills magma calculated. The color gradient in the figure represents sample distribution density, with upper continent crust and Iceland rhyolite ranges included for comparison [38,55].

As shown in Fig. 2a, the recovered SiO_2_ content vary continuously from ∼57.8 to 78.1 wt%, with an average of 70.7 ± 3.6 wt%. The total alkali content (K_2_O + Na_2_O) varies from 4.0 to 8.5 wt% (Fig. 2a), with K_2_O/Na_2_O ranging from 0.1 to 1.2. The other major element components P_2_O_5_, MgO, TiO_2_ and Fe_2_O_3T_ also span a wide range (Fig. S10). These recovered major oxides show co-variations, with SiO_2_ being comparable with that observed for natural rock samples (Fig. S10). As shown in Fig. 2c, the parental magma of JHZs is slightly enriched in LREE relative to middle rare earth elements (MREE) and HREE, with depletions in Ti, P and, more pronouncedly, Nb. The patterns are similar to those of the average upper continental crust (Fig. 2c), yet they are distinct from those of Icelandic rhyolites [38,39], which exhibit less fractionated REE patterns and no negative Nb depletions. The Sr/Y and Eu/Eu* ratios of the recovered parental magma are 0.8 to 102.6 (mean of 16.3), and 0.1 to 2.7 (mean of 0.9), respectively (Supplementary Data).

### Diverse rocks and diverse sources

Firstly, our results show that the parental magmas of JHZs are not andesitic, but significantly more felsic, which is in contrast to the previous estimates (55–65 wt%) based on the (Th/Y)_melt__–_SiO_2_ correlation [4,8,20]. We note that our JHZs datasets have contained all zircon data from Turner *et al.* [8], which indicates that this difference could not be attributed to data bias. To evaluate whether this discrepancy arises from methodological differences, we performed a comparative analysis of SiO_2_ contents obtained from both approaches, using testing dataset samples that were reserved exclusively for validation and never involved in the development of our ML models. The analytical results demonstrate that our ML model can accurately reconstruct SiO_2_ contents within a 20% deviation range relative to reference values, whereas the Th/Y method yields significantly more variable SiO_2_ estimates (Fig. S11). The deviation of the latter method would arise from the largely overlapping Th/Y ratios for global andesitic and high-SiO_2_ rocks (Fig. S11), which can result in a wide range of recovered SiO_2_, especially when sparse Th/Y data (e.g. recovered from detrital zircons at a specific age) were used. However, discussing how to successfully apply the Th/Y method is out of the scope of this work.

The big advantage of our ML model is that it can also provide constraints on many other major elements. We further infer the rock types of the parental magma for JHZs with consideration of multiple oxides content. On the total alkali (Na_2_O + K_2_O) vs. SiO_2_ diagram, they can be identified as rock series from diorite through granodiorite to granite (Fig. 2a). In the K_2_O vs. SiO_2_ diagram, most of the rocks fall into the medium- to low-K series and overlap with the Archean TTG field, with some of them also plotting in the field of potassic granites (Fig. 2b). This identification is consistent with the consideration in the aspects of K_2_O/Na_2_O ratio [24,25,40,41] (the K_2_O/Na_2_O ratio of TTG is <0.6; 58% of JHZs are this type) and the An–Ab–Or diagram [42], which are based on the normative mineral compositions calculated from the recovered major element compositions of parental magma (Fig. S9a). These results suggest that the types of the source rocks for the JHZs would be rather diverse, varying from andesitic to granitic in lithochemistry, and from TTGs to potassic granites in lithology.

**Figure 3.**
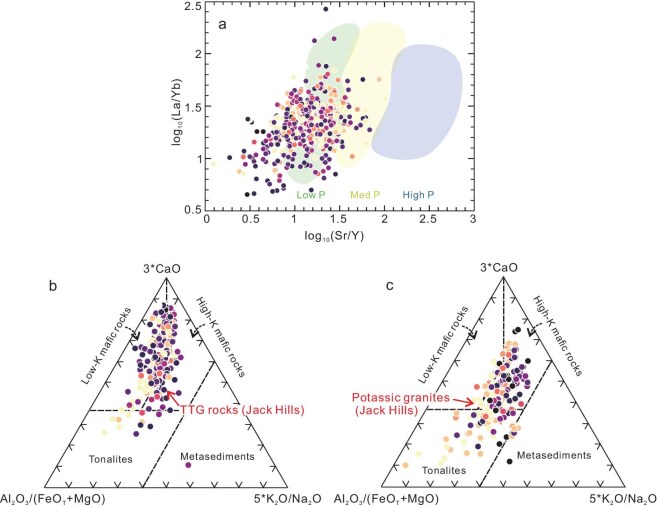
Pressure types and potential source rocks of the Jack Hills ‘rocks’. (a) Log_10_(La/Yb) vs. log_10_(Sr/Y) diagram [26]. The high-, medium- and low-pressure (High P, Med P and Low P) TTG ranges in the diagram are based on Moyen [27]. (b) Ternary diagram Al_2_O_3_/(FeO_T_ + MgO) vs. 3*CaO vs. 5*(K_2_O/Na_2_O) [40]. This diagram is used to identify potential source rocks for TTG rocks. (c) Ternary diagram of Al₂O₃/(FeO_T_ + MgO) vs. 3*CaO vs. 5*(K₂O/Na₂O) for identifying potential source rocks of potassic granites. The sample symbols are consistent with those in Fig. 2.

It is critical to identify the role of either fractional crystallization of mantle-derived mafic magmas or partial melting of the mafic crust, because both processes can produce the continental crust of felsic composition [43]. In this work, both the recovered TTGs and granites exhibit relatively low Sr/Y ratios (<105, mainly lower than 50) and negative Eu anomalies, suggesting the possible role of plagioclase via crystal fractionation or partial melting at shallow depths. However, the absence of correlations for Na_2_O vs. Sr/Y, SiO_2_ vs. Eu/Eu*_N_, and MgO vs. (Dy/Yb)_N_ (N represents chondrite normalization) plots and the positive correlation between La/Sm and La rules out the significant fractionation of plagioclase and amphibole [44,45] (Fig. S12). Thus, Sr/Y ratios can be used to infer the formation depth of parental magmas. Plotting the calculated whole-rock compositions on the log_10_(Sr/Y) vs. log_10_(La/Yb) diagram reveals that the majority of parental magma belongs to the low- to medium-pressure TTG series [26] (Fig. 3a). We also note that the recovered (Dy/Yb)_N_ ratios of our TTG and potassic granites may be up to >2, but the average value and range totally overlap with that of the low- to high-pressure Archean TTG and potassic granites (Fig. S13), respectively. Overall, our results show that the parental magma produced by high-pressure partial melting was absent for the JHZs.

Below, we constrain the potential sources of parental magma for JHZs. On the MgO vs. SiO₂ diagram (Fig. S9b), these samples fall within the partial melting range of tholeiitic basalt and display low MgO + FeO_T_ content (mostly <10 wt%, with a mean of 5.0 wt%), indicating that they would originate from a pure crustal source [40]. Based on the Al_2_O_3_/(FeO_T_ + MgO) vs. 3*CaO vs. 5*(K_2_O/Na_2_O) ternary diagram (Fig. 3b and c), which has been widely used to identify the source compositions of Archean granitoids [40], it is shown that these TTG magmas were mainly derived by partial melting of low-K mafic rocks, with a small portion of them sourced from tonalite. On the other hand, the potassic granites were mainly sourced from high-K mafic rocks, with a portion of parental magmas (with age from 4.2 to 3.3 Ga, especially for those later than ∼3.6 Ga) produced by partial melting of the pre-existing tonalite (Fig. 3b and c).

**Figure 4.**
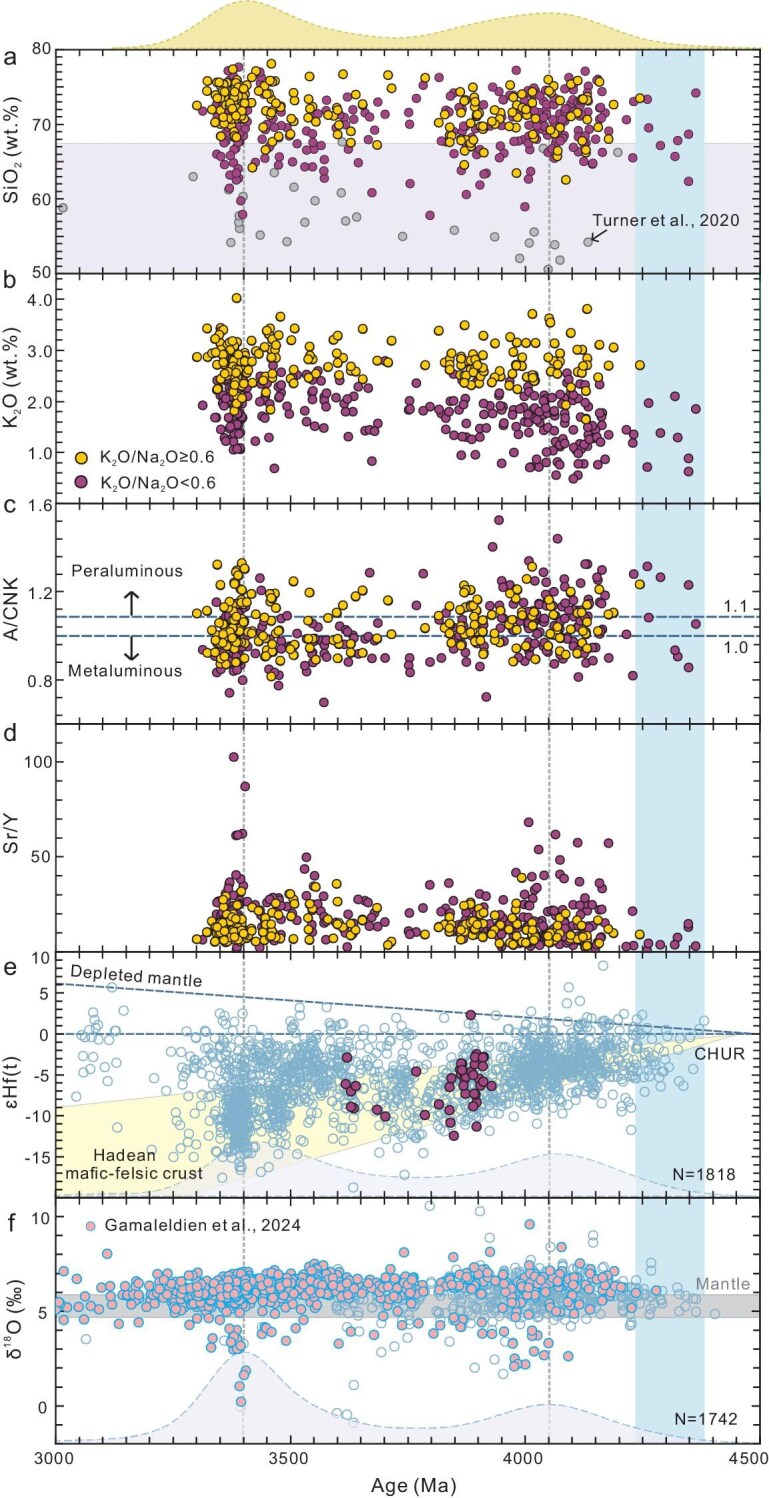
Secular evolution of geochemical composition in Jack Hills ‘rocks’. Correlations of (a–d) the major and trace elemental ratios for the parental magma from two periods at 3.4 and 4.1 Ga. (e–f) Previously published zircon Hf and O isotopic data were plotted [6,16–18,20,46,58–62]. (e) The filled and open dots are the zircons with and without oxygen isotope data, respectively. The filled dots in (f) are the data from ref. [46].

For both types of parental magmas, metasediments were not the direct source (Fig. 3). This is in contrast to the previous suggestion that more than 40% of the parental magmas for JHZs were derived from recycled sedimentary rocks [21–23]. This discrepancy would arise from the previous classification models that determine the source on the basis of aluminum saturation, relying on the discrimination between I-type and S-type granites. However, the values of the aluminum saturation index (calculated as A/CNK) overlap, even for Archean TTG and potassic granites [27,40] (Fig. S14). The observation that our TTG and potassic granites show a comparable range of A/CNK (Fig. 4) implies that the enrichment of K in the TTG source is decoupled from aluminum, and indeed does not support a significant sedimentary contribution.

### Implications for the origin of felsic crust on early earth

As shown in Fig. 4, during the first 250 Ma, nearly all the recovered rocks are identified as TTGs according to their low K_2_O/Na_2_O ratio, with recovered Sr/Y ratios lower than 15. The δ^18^O values of the other JHZs in the literature (without trace element data, thus not applied in our ML model) are almost within the generally accepted range of normal mantle. Potassic granites have coexisted with TTGs since their initial occurrence at around 4.2 Ga (Fig. 4b). Their Sr/Y ratios vary from 1.3 to 39.0 (mostly <25) and do not change considerably (Fig. 4d), implying partial melting at relatively shallow depths. At time intervals centered at ∼4.0–4.2 and ∼3.4 Ga, the peaks for K_2_O and SiO_2_, as well as Sr/Y ratios, can be identified for TTGs (Fig. 4a–d). These peaks also correspond to the highest age distribution density and the largest deviation in δ^18^O compared with the normal mantle, especially corresponding to the time of occurrence of extremely low δ^18^O values that may reflect the hydrological cycle of surface water [46]. Overall, our ML results reveal that the parental magma of JHZs were not dominated by andesitic rocks, but by felsic rocks, including TTGs and potassic granites, which were produced by the partial melting of mafic rocks with varying degrees of enrichment in melt-mobile incompatible trace elements.

The recovered primitive mantle-normalized trace element patterns for the parental magma of the JHZs are distinct from that for the rhyolite in Iceland (Fig. 2c), which rules out the Iceland-like mantle plume model [12,13]. The heat-pipe tectonics model has also been proposed to be the main geodynamic regime on early Earth [47]. However, numerical geodynamic models suggest that a volcanism-dominated heat-pipe tectonics model cannot account for the primordial crust with the observed proportions of various TTG compositions, whereas the intrusive magmatism, generating hotter crustal geotherms, can produce the diverse TTG rocks [48].

The petrogenesis of Archean TTGs may provide a substantial reference to that of Hadean felsic crust. The popular presence of high-pressure TTG (Sr/Y > 110; >1.5 GPa), large portion of andesite, and the arc-like trace elemental patterns (e.g. depletion in Nb and Ta relative to La) have been suggested to be consistent with the production of TTG magmas through partial melting of subducting oceanic slabs at modern-like subduction zones [8,26,27,41,49]. Sotiriou *et al.* [50] summarized the temporal variations in the incompatible trace element systematics of Archean TTGs, and suggested that most Archean TTGs show adakitic characteristics (high La/Yb and Sr/Y ratios) and were formed by partial melting of subducting oceanic crust or the lower arc crust under modern-style plate tectonic processes. This aligns with the previous geological studies, which proposed that subduction beneath arc systems was already active during the Eoarchean [51,52]. For the recovered parental magma of JHZs, the Sr/Y ratios are all lower than 110, mostly ranging from 0.8 to 68.3, suggesting that the associated partial melting occurred at low to medium pressures (Sr/Y ≤ 110; <1.5 GPa) [25,27,41,53]. This result is consistent with previous pressure estimates obtained from primary phengitic mica inclusions in JHZs (0.5–1.2 GPa) [10]. The absence of high-pressure TTGs and the presence of only a small proportion of andesite in the JHZs’ parental magmas differ from the general characteristics of typical Archean TTG suites. Thus, if the partial melting of subducting oceanic slab usually applied for Archean TTG rocks is applicable for the parental magma of JHZs, our results imply that the melting occurred at relatively shallow depths.

Zheng *et al.* [49] proposed a three-stage model for the petrogenesis of Archean TTG with the framework of microplate tectonics. In this model, the basaltic oceanic crust was generated at divergent plate margins and then transported to convergent plate margins, where it could be not only subducted at low angles but also collided for significant thickening. The subsequent thinning of the lithospheric mantle beneath the thickened crust would induce upwelling of the asthenospheric mantle, heating the overlying mafic crust to cause partial melting and generate TTG magmas, which would eventually ascend and intrude into the overlying rocks due to their buoyancy. If this petrogenetic model of Archean TTG rocks is applicable to the Hadean felsic crust, it implies the operation of convergent tectonics in the Hadean. As shown in Fig. 4, during the two periods of ∼4.0 and ∼3.4 Ga, the Sr/Y ratios vary widely, and the diversity of both major and trace elements expanded. This observation is consistent with various pressure and temperature conditions for partial melting of the oceanic crust in the model of Zheng *et al.* [49]. In this scenario, it is possible for the continental crust on the Hadean crust to be produced primarily by partial melting of the collisionally thickened oceanic crust at previously convergent plate margins. Regardless of which of the two models discussed above better reflects the actual conditions of the Hadean, our results at least demonstrate that felsic continental crust could have formed at convergent plate margins during the Hadean, although the scale of such convergence cannot be directly constrained by our data.

Nevertheless, there are several aspects of the limitations in our work for the reconstruction of Hadean crustal compositions: (i) although random sampling and cross-validation were adopted to promote statistical independence, from a geological standpoint the training set still contains multiple zircon grains extracted from the same rock sample, which would weaken the assumption of independent and identically distributed data for regression and may possibly lead to an overestimate of model performance [54]; (ii) while our ML model demonstrates robust predictive power in estimating whole-rock major elements from zircon trace elements, its inherent ‘black-box’ nature introduces limitations regarding physicochemical interpretability. This limitation would possibly reduce confidence in predictions when applied to unconventional zircon compositions or novel tectonic settings; (iii) the proportion of the JHZs having trace element and other isotope data are still small; and (iv) on a global scale, Hadean zircons from many other locations were reported [2], but have not been extensively studied like the JHZs.

As the number of analyzed samples grows, this limitation can be mitigated by expanding the diversity of host rock types and increasing the number of rock samples and zircons per type, thereby generating more comprehensive datasets for training models with enhanced accuracy and robustness. In addition, other data pre-processing strategies, such as taking the average or median value of zircons within each rock, could be tested. For the second issue, future work should integrate explainable AI techniques to deconstruct the model's predictions into geochemically meaningful contributions. Moreover, coupling these data-driven insights with experimental petrology (e.g. high-pressure/high-temperature crystallization experiments) or thermodynamic modeling could help validate whether the identified correlations align with established fractionation processes or indicate novel partitioning behaviors. For the latter two limitations, if they do not violate the general features of the reconstructed parental magma for JHZs, our inference on the origin of continental crust on the Hadean Earth can stand under close scrutiny. In order to constrain the origin of the continental crust on early Earth more convincingly, a series of comprehensive studies on the global Hadean and Archean zircons should be conducted at the levels of both element and isotope compositions.

## Supplementary Material

nwaf230_Supplemental_Files

## Data Availability

All computational source codes and data used in this paper are available on the GitHub repository (https://github.com/ZJUEarthScienceJIA/Zircon_ML_Model_LDG).
