# Supplementary material for: Earth's Hadean crust formed via operation of convergent tectonics
Source: Natl Sci Rev. 2025 Jun 2;12(8):nwaf230. doi: 10.1093/nsr/nwaf230 (PMC12392895; doi:10.1093/nsr/nwaf230)
Supplement: nwaf230_Supplemental_Files [file nwaf230_supplemental_files.zip › Supplementary Information-Jack Hills-0528.pdf]

Supplementary Information for

"Earth's Hadean crust formed via operation of convergent tectonics"

Denggang Lu[1], Jia Liu[1*], Qunke Xia[1], Zhikang Luan[1], Jingjun Zhou[1], Tianting Lei[1], Lu Wang[2], Eero Hanski[3]

[1] Research Center for Earth and Planetary Material Sciences, School of Earth Sciences, Zhejiang University, Hangzhou 310058, China

[2] Key Laboratory of Silicate Cultural Relics Conservation, Shanghai University, Shanghai 200444, China

[3] Oulu Mining School, University of Oulu, Oulu 90014, Finland

Corresponding authors:

Jia Liu, E-mail: liujia85@zju.edu.cn

This PDF file includes:

Supporting text

Data compilation and filtering

Machine learning modeling

Key Terminology Explanation

Figures. S1 to S18

Tables S1 to S2

References

Supporting text

Data compilation and filtering

Global geochemical datasets of zircon (36277) were compiled from the GEOROC database (http://georoc.eu/) and through bibliographic retrieval. We carefully checked the age and element contents of the zircons, selecting 25750 valid zircons based on the methodology by Wang et al. (2024)[1]. The whole-rock data for these filtered samples were matched, resulting in 14241 zircons paired with 821 whole rocks (Supplementary material). These samples cover a wide geochemical range (Supplementary Figure S2). We followed by checks for whole-rock alteration (Supplementary Figure S16) and zircon metamorphism (using CL, transmitted/reflected microscopy, original alteration descriptions, age, and data accuracy where possible). After rigorous data screening of the 14241 zircons, excluding the influence of metamorphism and other factors, we obtained effective data for 9879 zircons and their matching whole rocks (LOI $\leqslant 4$; 6759 data points with LOI ≤ 0.2), including 9454 zircons with ages. All zircon data were filtered using the following criteria to ensure the original magmatic composition[2–4]: (1) LREE-I $\geqslant 30$, (2) La $\leqslant 0.5$ ppm, (3) Ca $\leqslant 150$ ppm and Fe $\leqslant 150$ ppm (if available), and (4) Th/U $\geqslant 0.1$. In addition, for ancient Archean whole-rock data, we assessed fluid mobility for key elements to ensure that subsequent analyses were not influenced by fluid alteration[5] (Supplementary Figure S16).

Machine learning modeling

Machine learning is primarily divided into regression models and classification models, with regression models quantifying the relationship between X and Y variables[6]. The construction of a complete regression machine learning model involves several key stages: data compilation and preprocessing, data splitting and model selection, and model training and evaluation (see the detailed modeling workflow in Supplementary Figure S17).

Before inputting data into the model, we first preprocessed and filtered the data based on prior geoscientific knowledge (refer to "Data Compilation and Filtering"). After preprocessing, we selected the trace elements and element ratios (Ti, Y, Nb, REE, Hf, Ta, Th, U, Th/Y, Dy/Yb, Nb/Th, Ce/Ce*, Eu/Eu*) of 9879 zircons as the input variables (X) for feature engineering, and the geochemical element data of the whole rocks as the output variables (Yi) (Supplementary material). We employed a result-oriented, machine learning–based feature selection strategy to retain key

geochemical information while reducing multicollinearity. This approach enhances model robustness and interpretability without relying on prior geochemical assumptions. In machine learning, datasets are typically divided into training and testing sets (e.g., 7:3, 8:2, or 9:1 ratios). Given the high dimensionality of our dataset (involving multiple (X) and (Y) variables), we tested different proportions and chose a 9:1 ratio for the training and testing sets to provide the machine learning model with more information. This ratio allows for maximum utilization of the available data for training, ensuring model stability and reducing overfitting.

For model selection, considering the common presence of missing values in structured geoscientific data, we needed a model that could tolerate such missing values while retaining sufficient effective information for machine learning. After comparing different models and adhering to the principle of result reliability in machine learning, we chose the XGBoost algorithm, based on gradient boosting decision trees, to establish the regression model[7]. Hyperparameters significantly influence the performance of a regression model. Fine-tuning them can enhance model performance and prevent overfitting. We used Random Search Cross-Validation from the sci-kit learn package to iteratively find the optimal combination of hyperparameters (max depth, learning rate, and alpha). Data imbalance can impact the generalization ability and overall performance of the model. We used 10-fold cross-validation on the training dataset to assess the overall performance of the model. The entire dataset of key elements was randomly split 1,000 times in a 9:1 ratio to observe the distribution of R^2 scores and further evaluate potential overfitting and model robustness (Supplementary Figure S18). This approach also ensures that the performance evaluation is not reliant on a single random split while addressing and eliminating potential data imbalance issues. The results showed that the scores for our test set were near the peak of the distribution (Supplementary Figure S18). Additionally, to fully assess the model's reliability and generalization capability, we tested four completely independent cases (external test sets), which were separate from both the training and test sets. The results also indicate good performance on the external test sets (Supplementary Figures S6-S8). This comprehensive approach ensures the reliability and robustness of our machine learning model.

Our final evaluation of data performance in predicting elements of the whole rock involved using RMSE and R^2 metrics. R^2 serves as an indicator of goodness-of-fit, measuring how well the model generalizes to unseen samples. The RMSE value represents the standard deviation of the

prediction error. The equations for calculating R^2 and RMSE are:

$$R^2 = 1 - \frac{\sum_{i=1}^{n}(Y_i - y_i)^2}{\sum_{i=1}^{n}(Y_i - Y)^2}$$

$$RMSE = \sqrt{\frac{\sum_{i=1}^{n}(Y_i - y_i)^2}{n}}$$

where Y_i and y_i represent the true and predicted values of whole rock respectively. Y represents the average true whole rock values of the samples, while n indicates the number of samples.

Key Terminology Explanation

10-fold cross-validation is a widely used technique in machine learning for model evaluation. It aims to provide a more reliable assessment of a model's performance by reducing bias caused by a single train-test split. The dataset is divided into 10 equal parts, where 9 parts are used for training and 1 part for testing. This process is repeated 10 times, with each part used as the test set once, and the final performance is averaged across all iterations. This method helps mitigate overfitting and ensures that the model generalizes well to unseen data.

The 1,000 random splits approach involves partitioning the dataset into training and testing sets 1,000 times, often using a 9:1 split ratio, to observe the distribution of performance metrics such as R^2 scores. By repeatedly training and evaluating the model across different splits, this technique allows for a robust analysis of the model's stability and its potential for overfitting, ensuring that performance is not highly dependent on specific data configurations.

XGBoost (Extreme Gradient Boosting) is a highly efficient and flexible machine learning algorithm widely used for tasks such as regression and classification. Its performance is heavily influenced by the careful tuning of hyperparameters. Key hyperparameters include:

1. **Learning rate**: Determines the contribution of each tree to the model. Lower values lead to slower convergence but may improve accuracy when combined with more boosting rounds.
2. **n_estimators**: Controls the number of boosting rounds (i.e., the number of trees). While more trees can increase model complexity, it also raises the risk of overfitting.

3. **max_depth**: Limits the depth of each tree, controlling the complexity of the model. Shallow trees help prevent overfitting, while deeper trees can capture more complex patterns.
4. **subsample**: Specifies the fraction of the training data used to grow each tree. Introducing randomness helps prevent overfitting by reducing variance.

Supplementary Figures

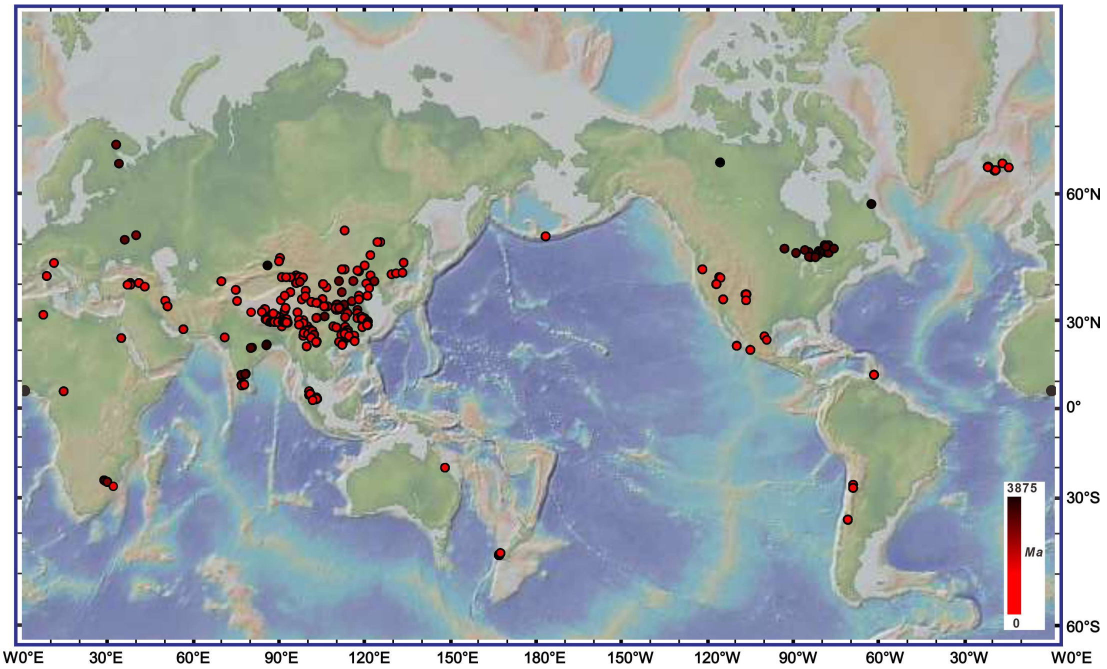

Fig. S1. Locations of samples in the machine learning dataset. Samples are shown as solid dots in the map, and color scale represents samples age (Ma).

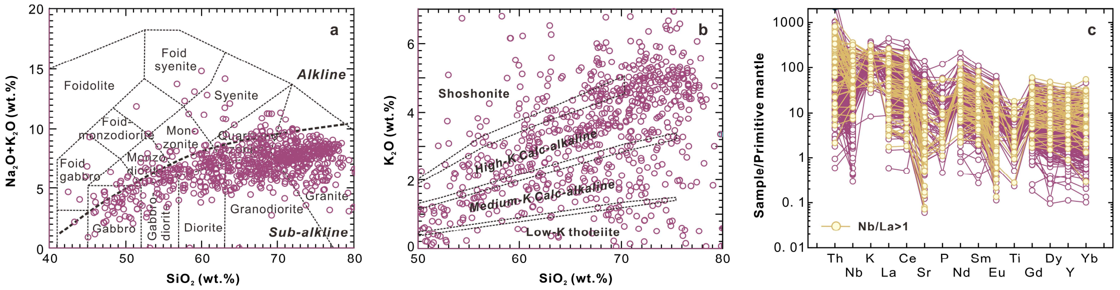

Fig. S2. Geochemical features of samples in the machine learning dataset. To understand the geochemical characteristics of the machine learning dataset, we plotted (**a**) Total Alkalis versus Silica (TAS) diagram[8], (**b**) K_2O-SiO_2 diagram[9], and (**c**) primitive mantle-normalized spider diagram[10] for 823 whole-rock samples matching with zircon trace elements. Samples with Nb/La ratios greater than 1 were individually marked in yellow. From the diagrams, we can see that our machine learning dataset covers a broad range.

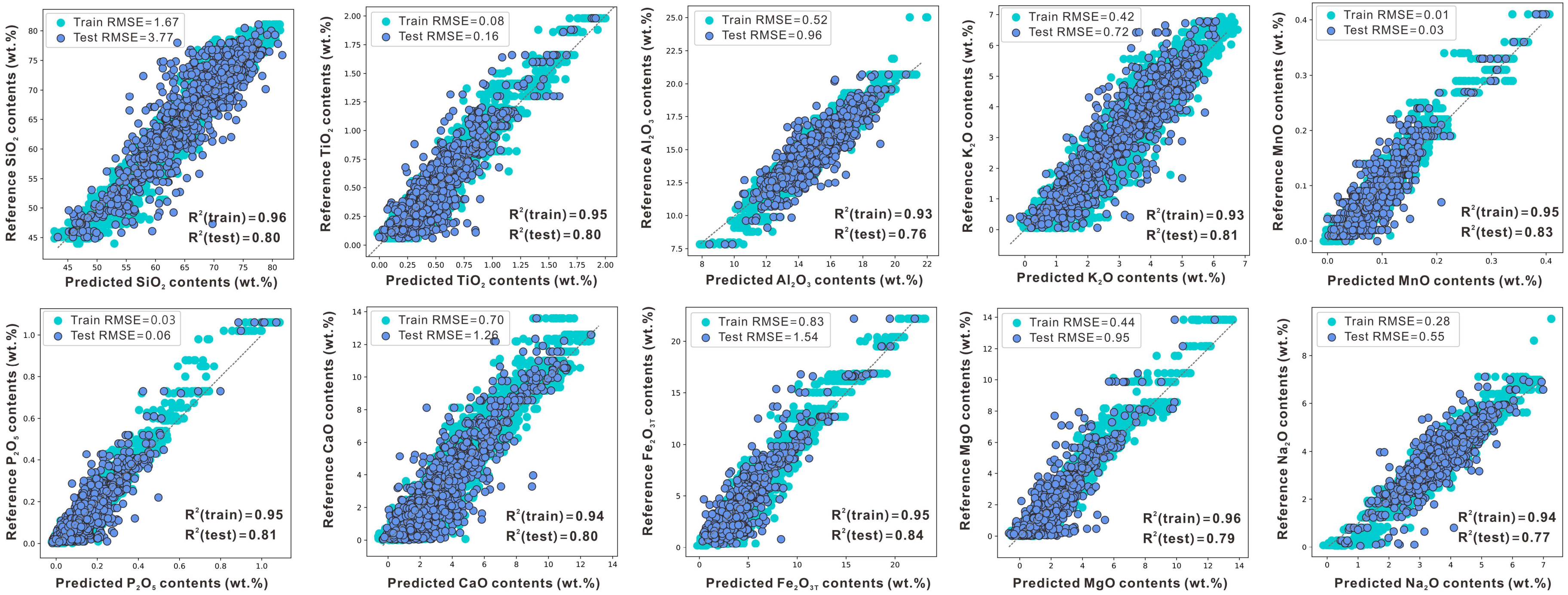

Fig. S3. Performance of Machine Learning Model. We constructed machine learning models for major elements using the XGBoost algorithm. The R^2 and RMSE scores for the testing set (blue dots) and training set (green dots) are depicted in the figure.

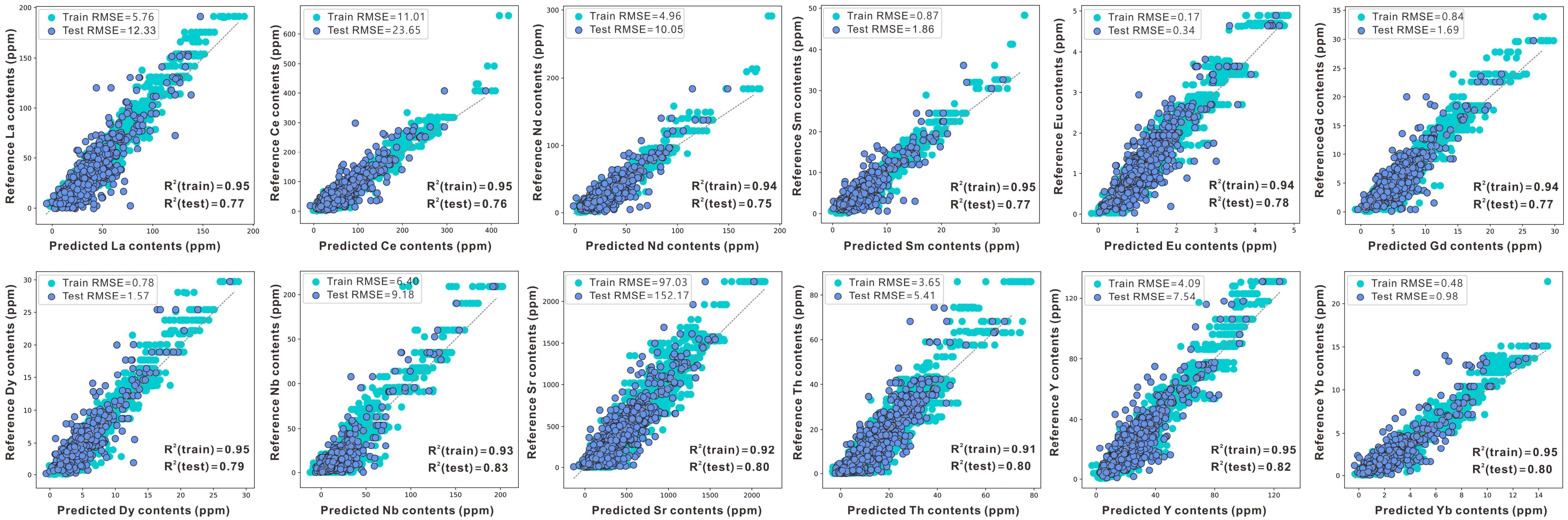

Fig. S4. Performance of Machine Learning Model. We constructed machine learning models for trace elements using the XGBoost algorithm.

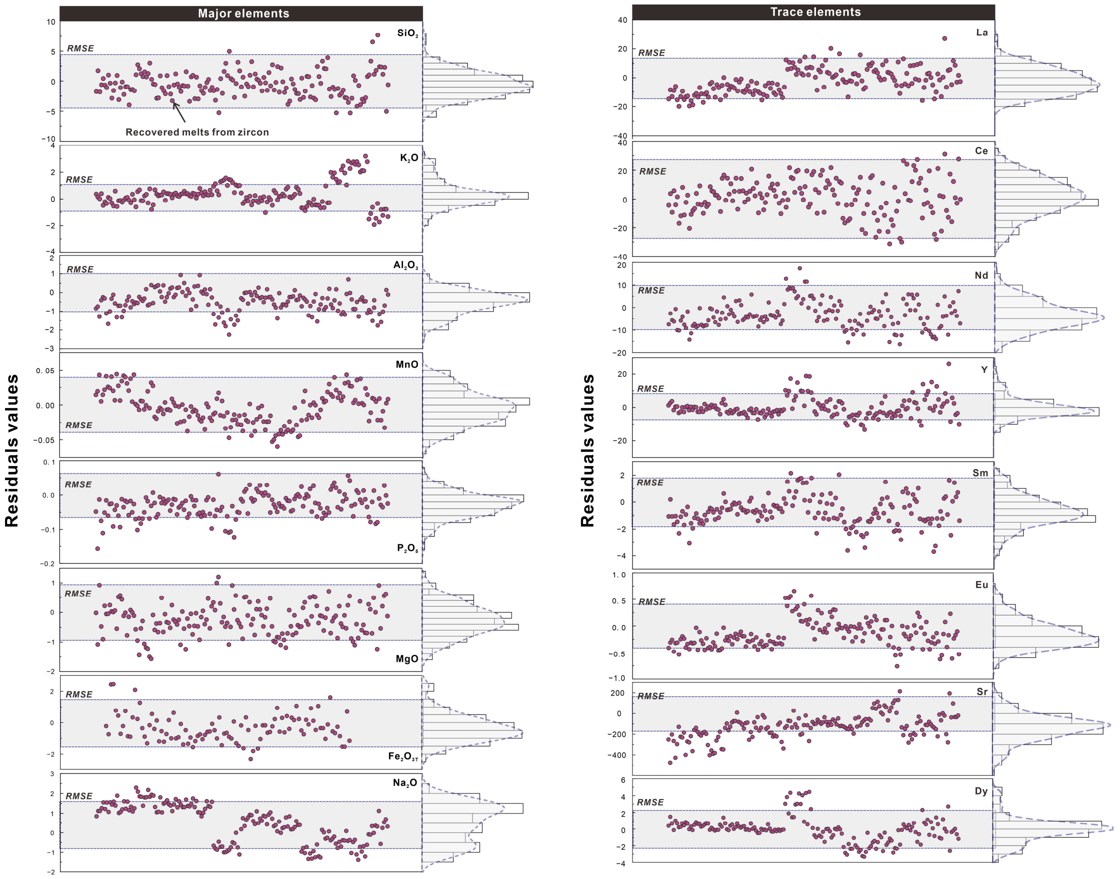

Fig. S5. Reliability of Machine Learning Model (Residual values). We selected three separate regions (not included in the training set) to test the reliability of our model. The residuals were computed as the predicted values subtracted from the true values. The gray shaded area represents the range of ±RMSE, which is also considered as the model's error. All major elements and key trace elements were tested.

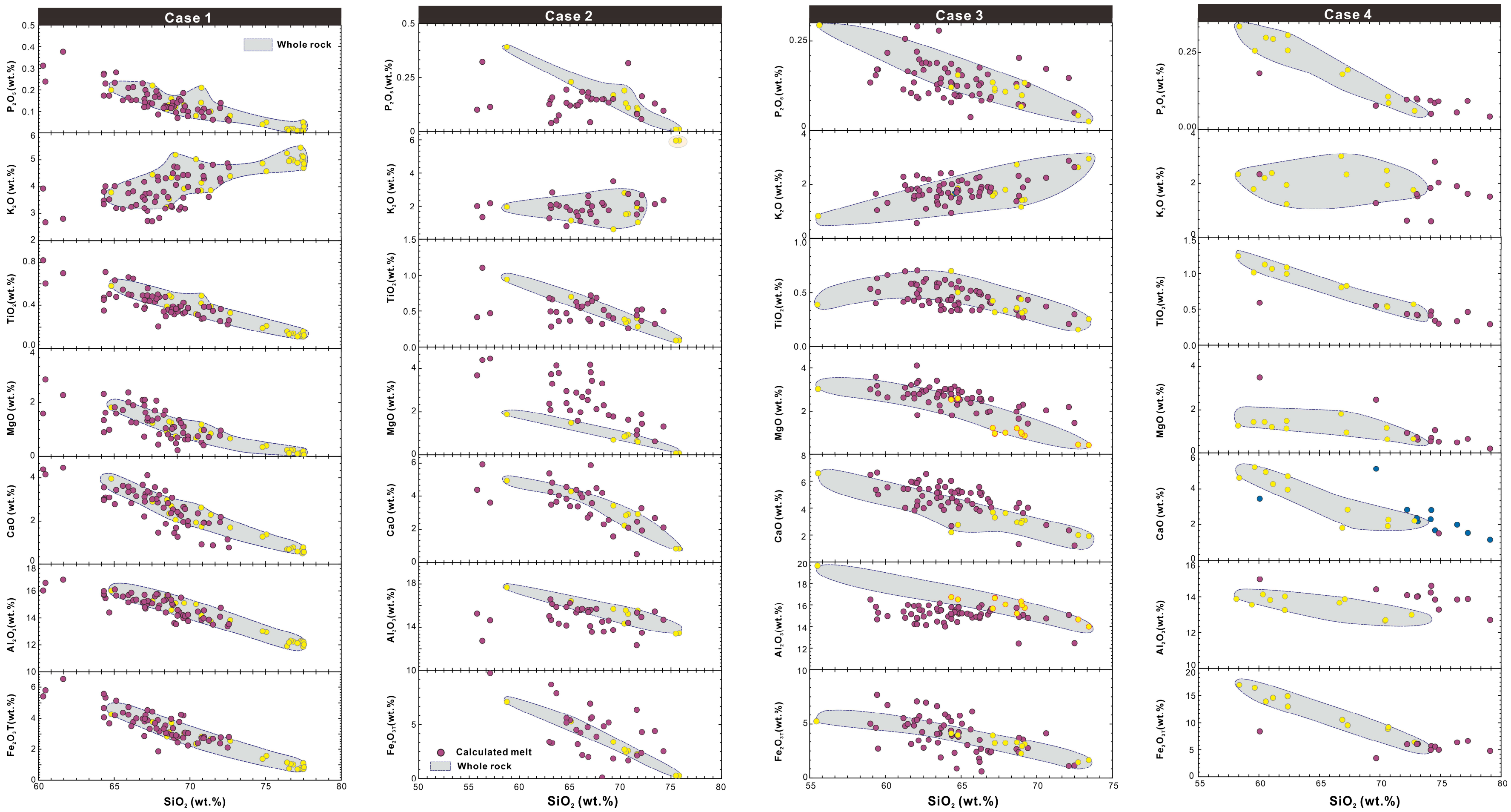

Fig. S6. Reliability of Machine Learning Model (Element correlations). Plots illustrating the relationships between major elements in four regions including the Cenozoic Gangdese belt, Archean Barberton, Tarim granites, and Hadean Acasta gneiss. They perform well within the margin of error.

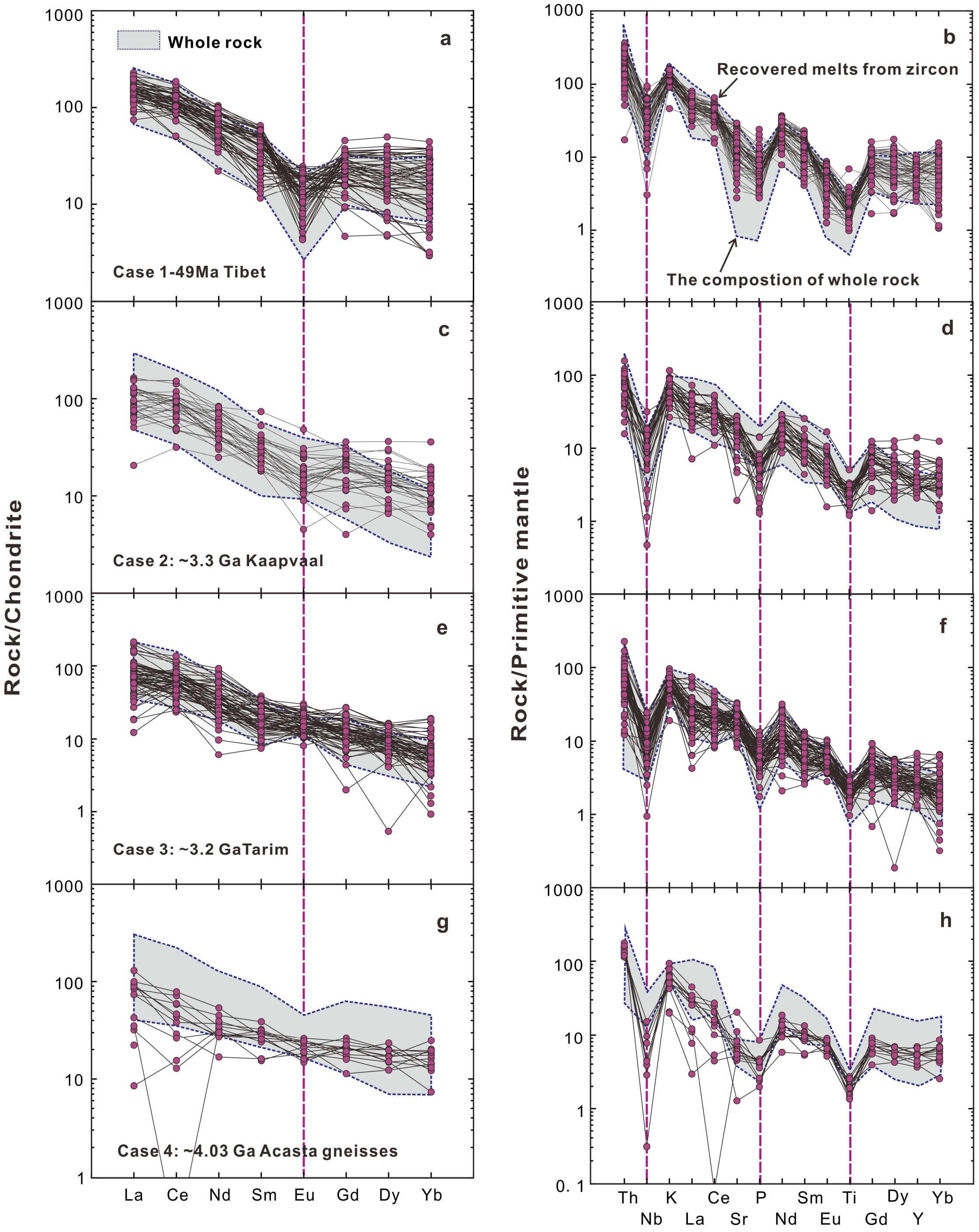

Fig. S7. Reliability of Machine Learning Model (Normalized diagrams). Chondrite-normalized rare earth element and primitive mantle-normalized trace element diagrams for four tested regions[3,11–13]. The chondrite and primitive mantle values are after Sun and McDonough (1989)[10]. They perform well within the margin of error, particularly for certain features such as Eu/Eu^{*}_{N} ratio and partitioning patterns. Symbols are the same as those in Fig. S6.

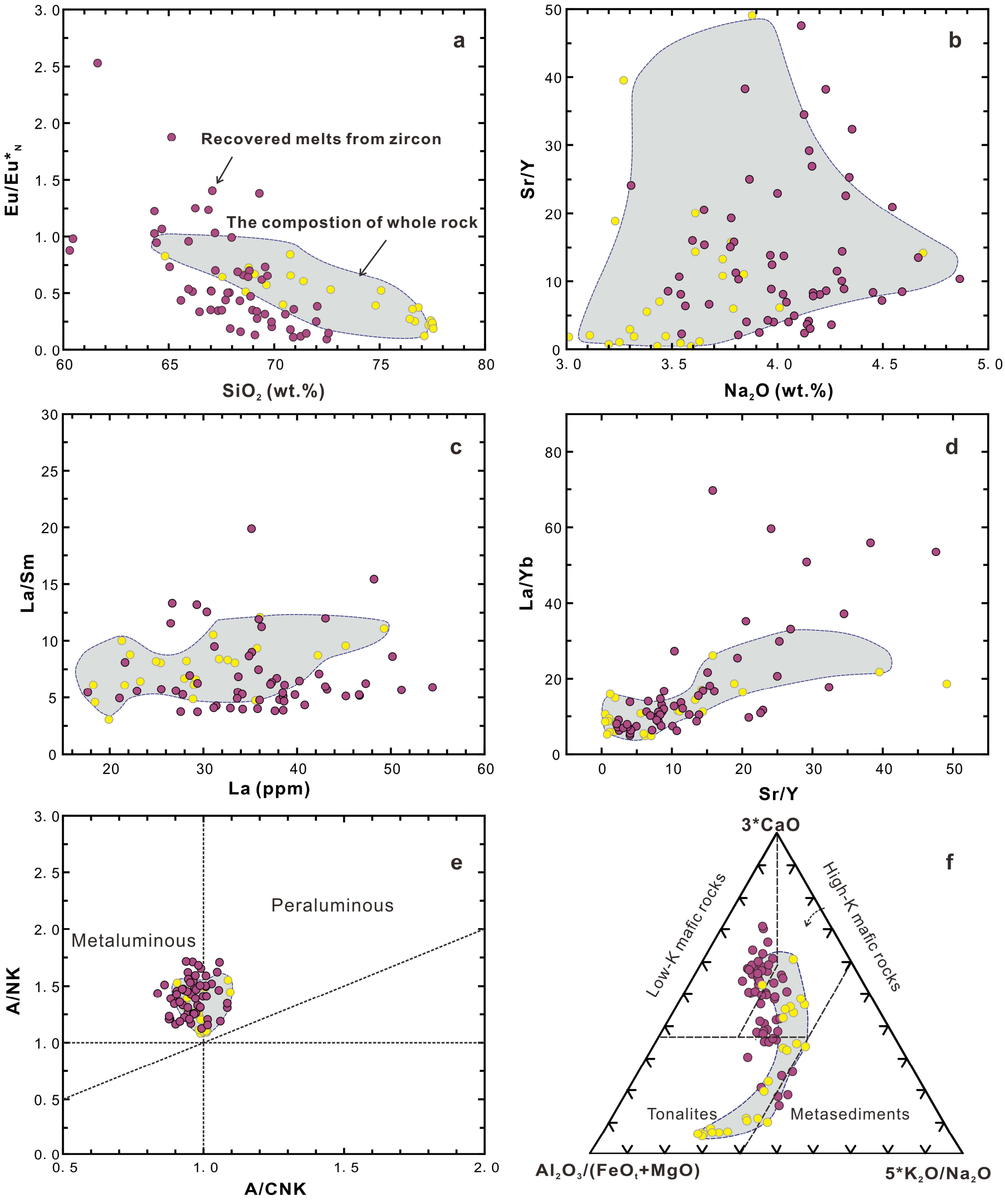

Fig. S8. Reliability of Machine Learning Model (Key element ratios). We conducted error assessments for some key geochemical parameters used in the main text, and the results indicate that within the margin of error, these geochemical parameters can effectively represent rock information. The data in the figure is sourced from Gangdese granites[12]. The citation of figures matches the citations in the main text.

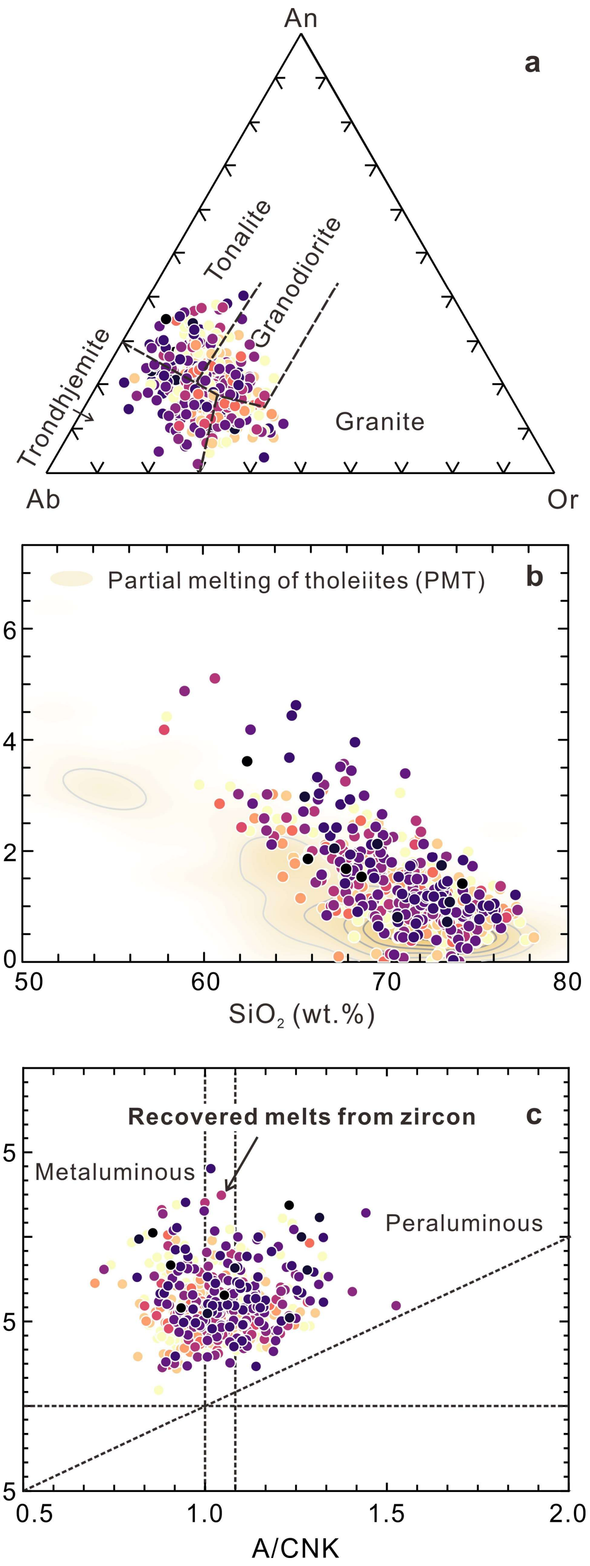

Fig. S9. Geochemical characteristics diagram of host rocks of Jack Hills detrital zircons. (**a**) An-Ab-Or diagram[14]. (**b**) MgO versus SiO_2 diagram (PMT: kernel density fields for experimental partial melts from tholeiites[5], and (**c**) A/CNK versus A/NK diagram.

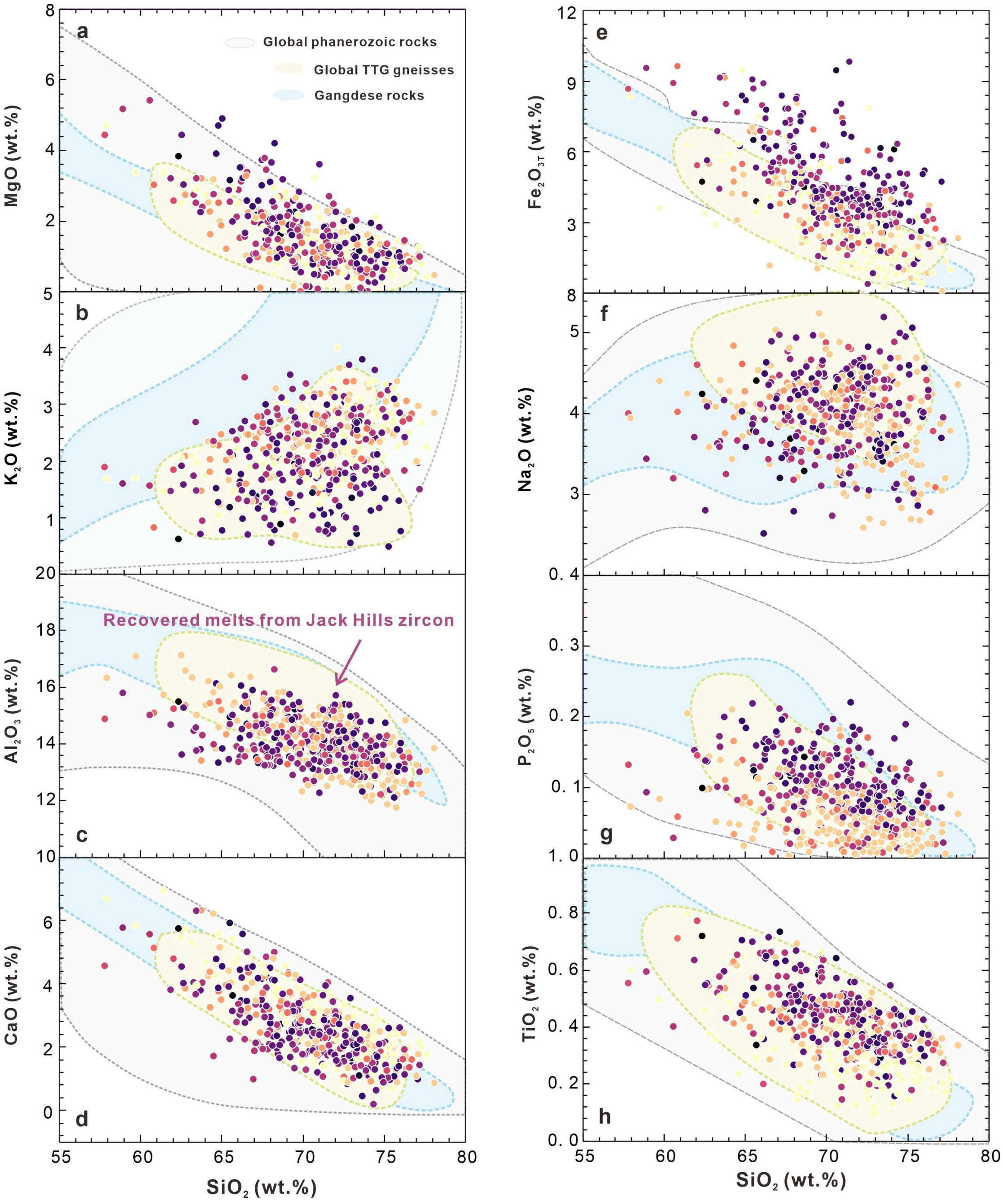

Fig. S10. Harker diagram of major elements of Jack Hills calculated parental magma. For comparison, we projected the ranges of Phanerozoic granites, Gangdese granites, and Archean TTG rocks. The yellow shaded area represents the range of global Archean TTG (Tonalite-Trondhjemite-Granodiorite)[15]. The blue shaded area represents the range of Gangdise granites (SiO_2 > 55 wt. %)[16]. The gray shaded area represents the range of global Phanerozoic granites (data from EarthChem database, http://portal.earthchem.org/).

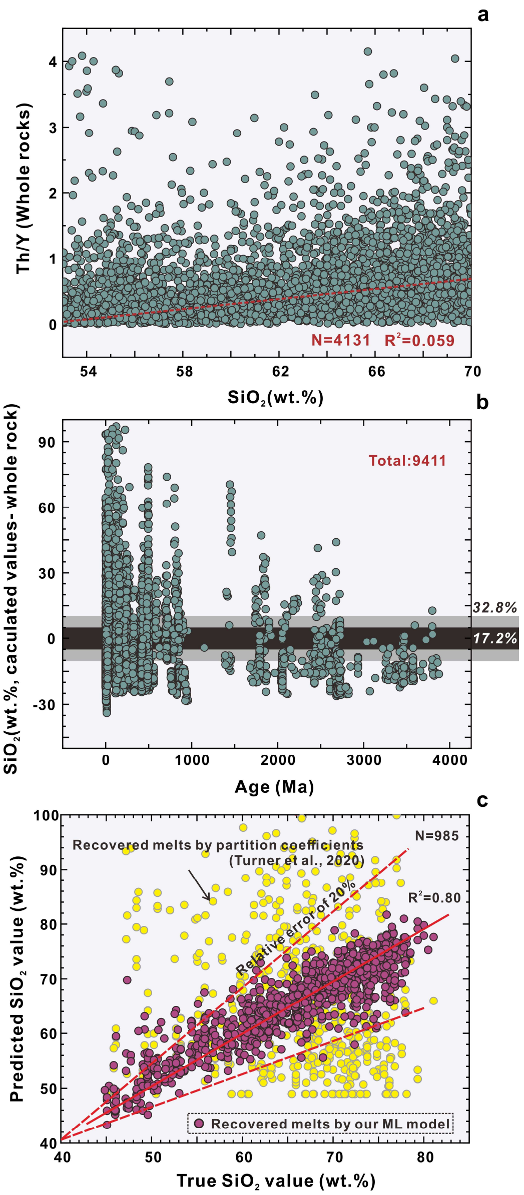

Fig. S11. The reliability test of the previous linear regression model. (**a**) SiO_2 vs. Th/Y diagram. Selecting 4131 valid data points from a total of 36667 data entries obtained from the global database (http://portal.earthchem.org/, EarthChem Download: Tuesday, Sep. 19, 2023 7:08:17 am) reveals no correlation between rock SiO_2 content and Th/Y ratio (R^2=0.059). (**b**) Residual SiO_2 vs. Age diagram. We tested the previous linear regression model using 9411 zircon data matched with their corresponding whole-rock data. The black shaded area represents the range where the calculated rock values deviate from the true values by less than 5%, with only 17% of the data falling within this range. The gray shaded area represents the range where the calculated rock values deviate from the true values by less than 10%, with only 32.8% of the data falling within this range. (**c**) Predicted value vs. true value diagram. The accuracy of melt SiO_2 reconstruction was compared between machine learning and previous partition coefficient methods. Dashed lines indicate relative errors of 20%.

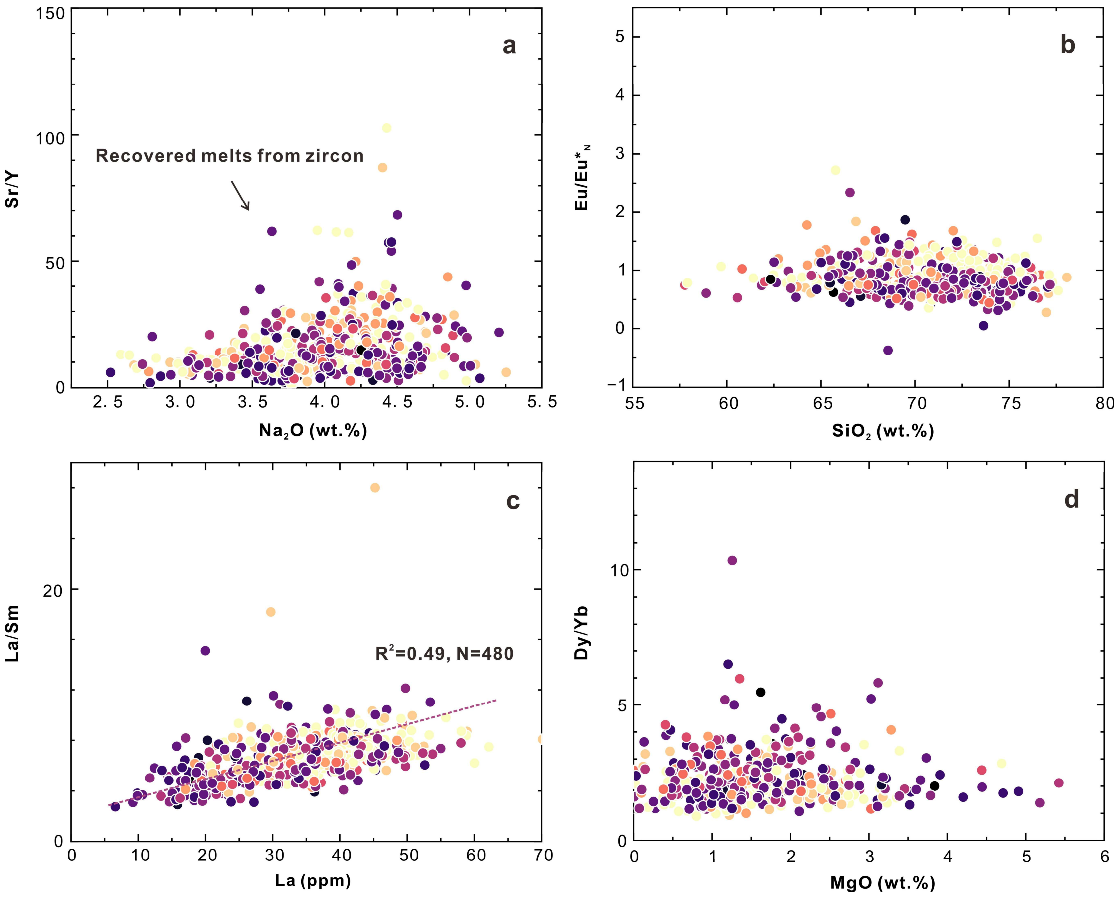

Fig. S12. Assessment of potential magma fractional crystallization processes. (**a**) Sr/Y vs. Na_2O diagram. (**b**) Eu/Eu^*_N vs. SiO_2 diagram. (**c**) La vs. La/Sm diagram[5,17]. (**d**) MgO vs. Dy/Yb diagram. The scatter plot colors represent ages, and color scale is the same as the color scale in the main text.

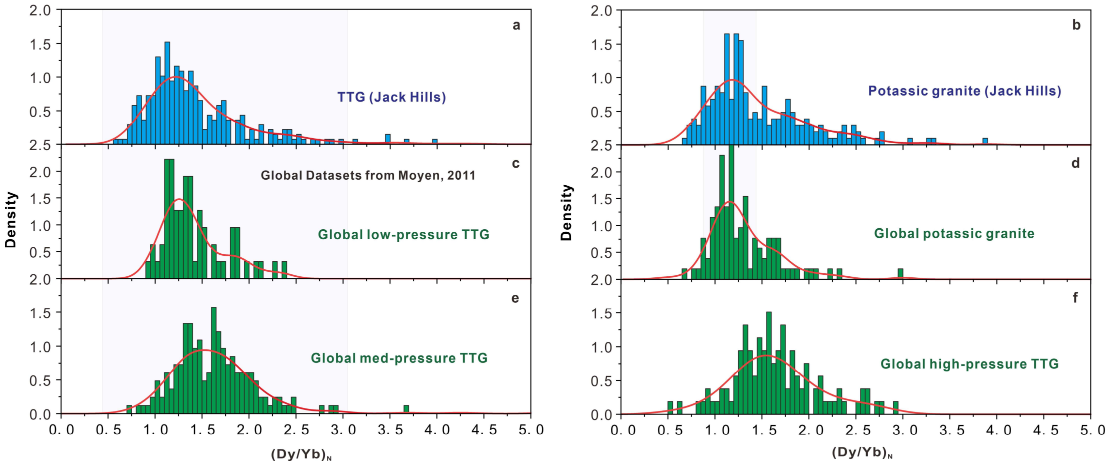

Fig. S13. Chondrite-normalized Dy/Yb kernel density histogram. The figure shows the chondrite-normalized[10] Dy/Yb ranges of high-, medium-, and low-pressure TTGs, potassic granites[15], and Jack Hills zircon parent magmas worldwide.

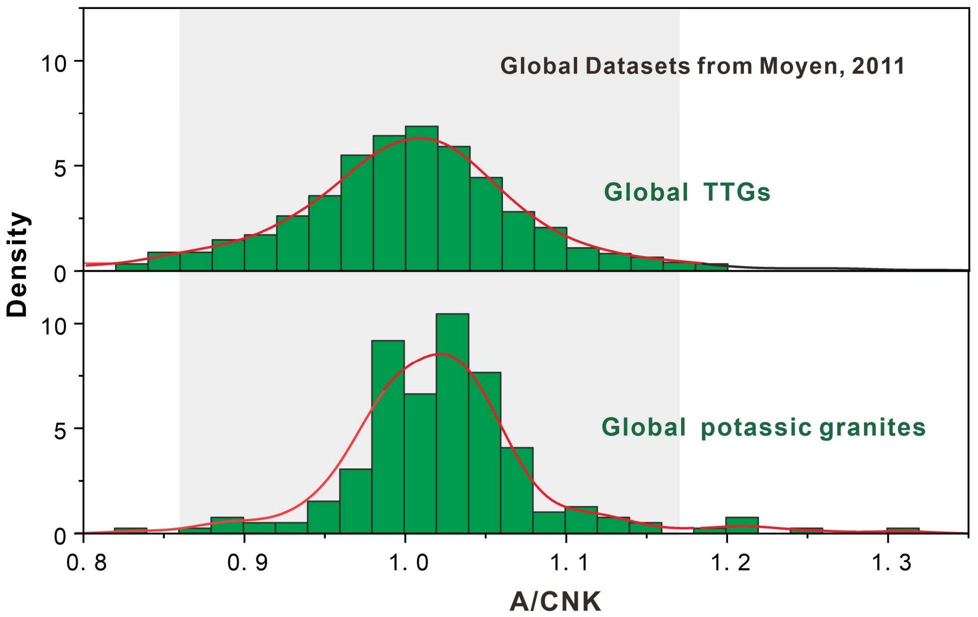

Fig. S14. A/CNK kernel density histogram of global TTGs and potassic granites.

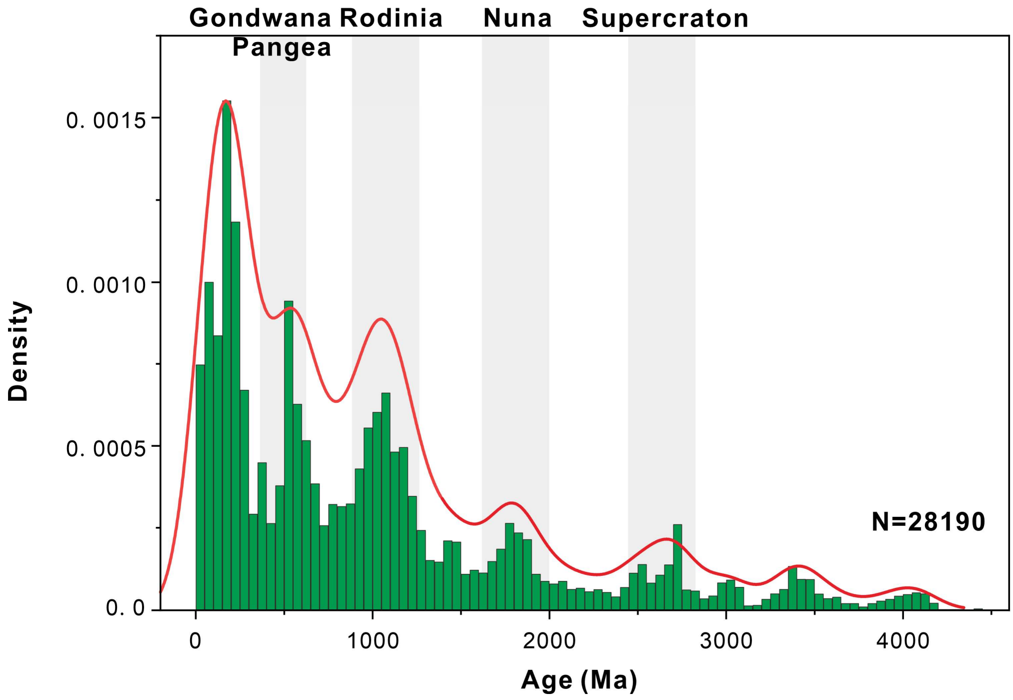

Fig. S15. Global kernel density distribution histogram of detrital zircons. The data for detrital zircons are sourced from Wang et al., 2024[1], with grey shading indicating several supercontinent events[18,19].

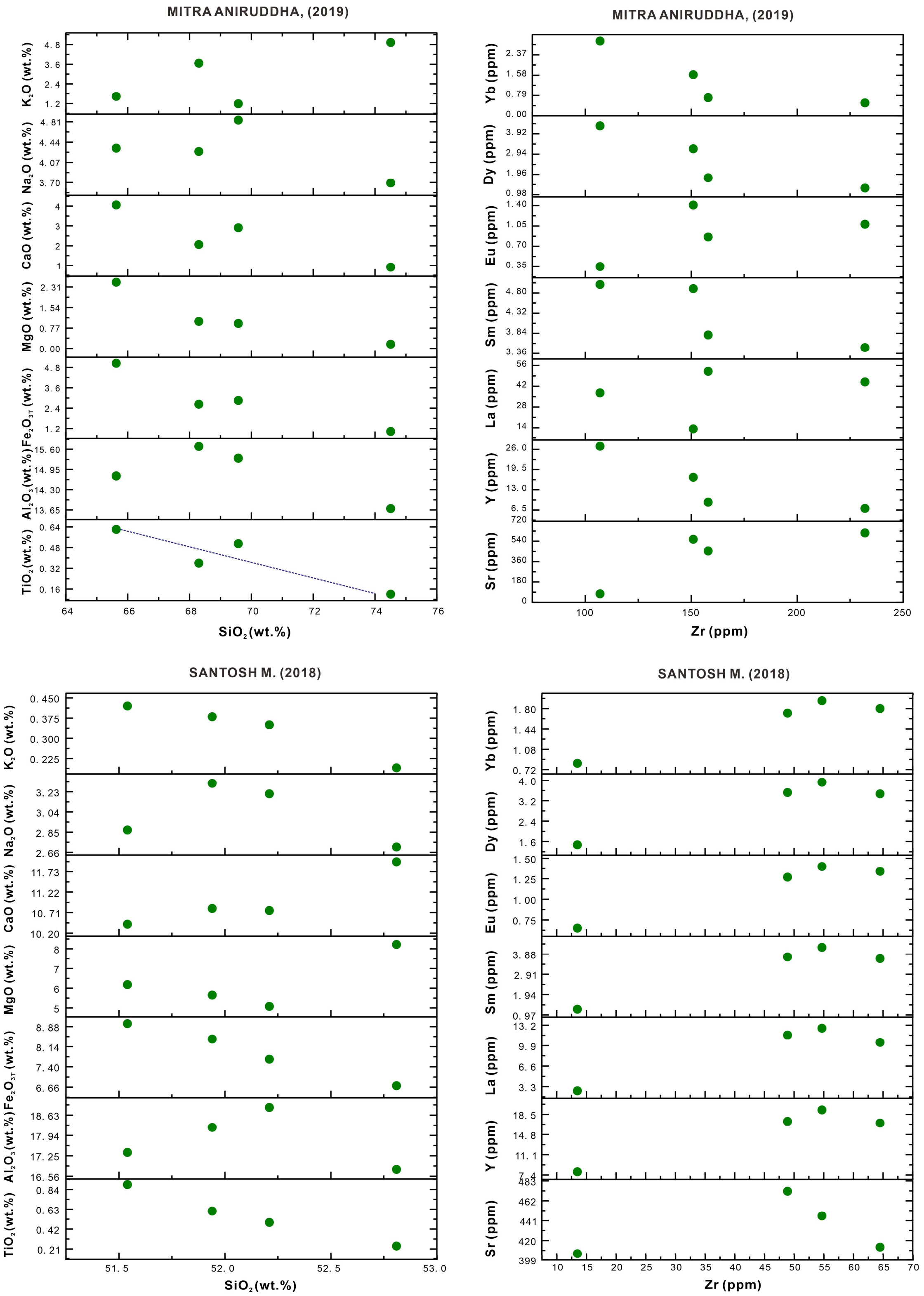

Fig. S16. Example of fluid-mobile element discrimination in global data collection. The good linear relationship displayed between the elements in the figure indicates that these elements have not been affected by later alteration.

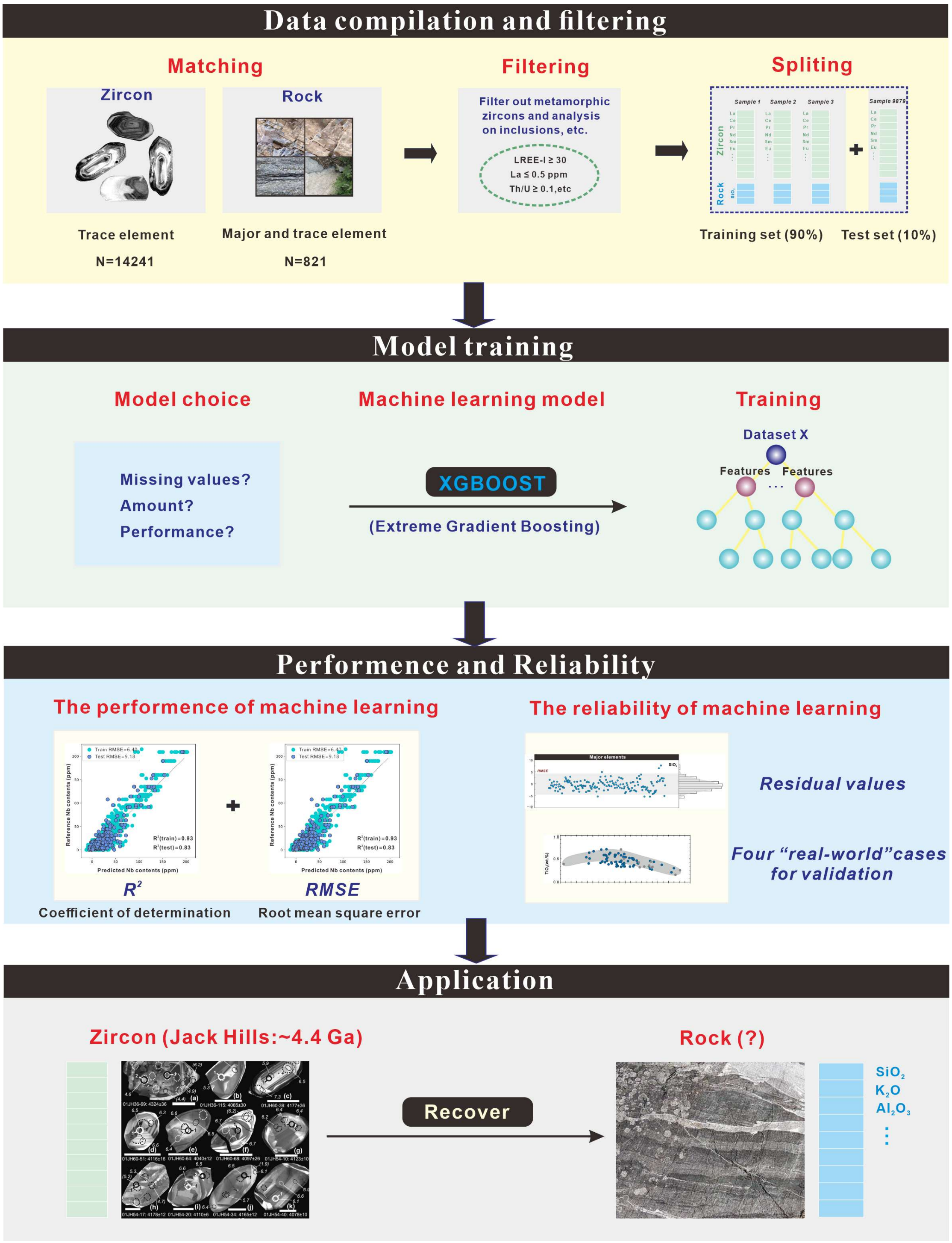

Fig. S17. The workflow diagram of the machine learning approach in this study.

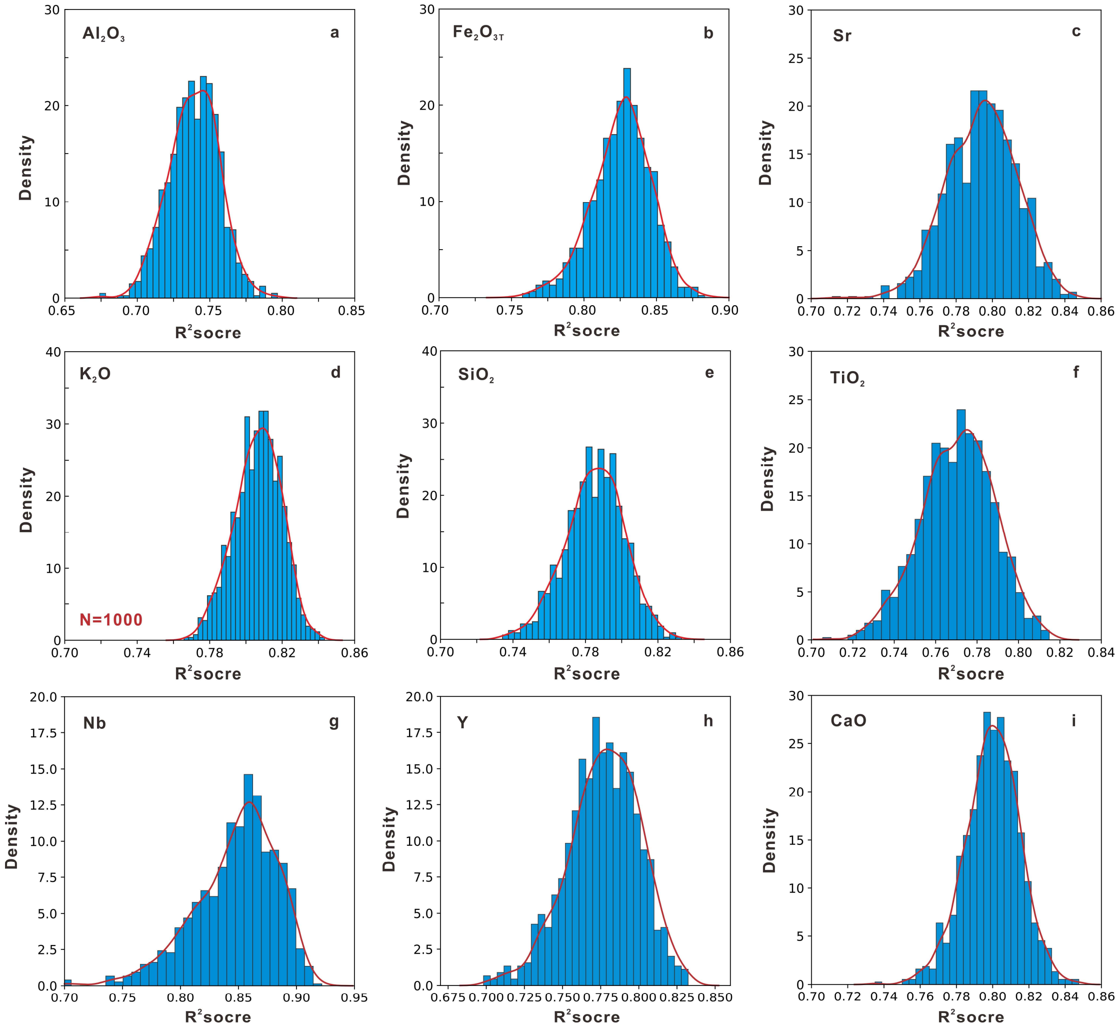

Fig. S18. The distribution of R^2 score for key elements in XGBoost machine learning. We trained and plotted the R^2 density distribution of the results from 1,000 training iterations on the complete dataset of key elements. It is observed that the R^2 score of the test set is located near the peak of the 1,000 distributions, indicating that the model is neither overfitting nor exhibiting good generalization capability.

Table S1. Performance of different regression algorithm models in 10-fold cross validation

Model		10-fold cross validation (Range)					
		R^2 (SiO_2)	RMSE (SiO_2)	R^2 (K_2O)	RMSE (K_2O)	R^2 (Fe_2O_{3T})	RMSE (Fe_2O_{3T})
**XGBoost**	Extreme Gradient Boosting	0.75-0.81	3.70-4.35	0.78-0.81	0.70-0.75	0.75-0.85	1.53-1.89
**RF**	Random Forest Regressor	0.58-0.73	4.22-5.22	0.71-0.81	0.68-0.87	0.56-0.79	1.60-2.09
**ANN**	Artificial Neural Networks	0.47-0.70	4.80-6.29	0.61-0.72	0.86-1.01	0.51-0.77	1.84-2.16
**KNN**	K Neighbors Regressor	0.55-0.69	4.85-5.74	0.62-0.75	0.82-1.02	0.51-0.76	1.82-2.23
**DT**	Decision Tree Regressor	0.25-0.49	5.81-7.04	0.45-0.72	0.89-1.23	0.45-0.66	2.03-2.86
**Ada**	AdaBoost Regressor	0.25-0.36	6.72-7.46	0.36-0.46	1.15-1.27	0.34-0.65	2.25-2.47
**Br**	Bayesian Ridge	0.09-0.18	7.09-8.81	0.23-0.43	1.26-1.45	0.10-0.25	2.87-3.58
**LR**	Linear Regression	0.10-0.18	7.17-8.79	0.23-0.43	1.25-1.80	0.07-0.26	2.97-9.16

Table S2. Performance metrics of the machine learning model: R^2 and RMSE.

	R^2 (Train)	R^2 (Test)	RMSE=Error (wt.%/ppm)	Range	10-fold cross validation (XGBoost)									
**SiO_2**	0.96	0.80	3.77	0.75-0.81	0.75	0.78	0.78	0.77	0.78	0.79	0.77	0.75	0.81	0.77
**Al_2O_3**	0.93	0.76	0.96	0.68-0.76	0.76	0.75	0.69	0.72	0.72	0.74	0.70	0.75	0.68	0.71
**Fe_2O_{3T}**	0.95	0.84	1.54	0.75-0.85	0.82	0.83	0.85	0.81	0.85	0.79	0.83	0.80	0.75	0.79
**CaO**	0.94	0.80	1.26	0.75-0.82	0.82	0.76	0.78	0.79	0.77	0.76	0.76	0.75	0.75	0.80
**MgO**	0.96	0.79	0.95	0.72-0.81	0.78	0.76	0.78	0.75	0.72	0.81	0.74	0.80	0.77	0.72
**K_2O**	0.93	0.81	0.72	0.78-0.81	0.80	0.81	0.81	0.80	0.79	0.80	0.81	0.78	0.78	0.79
**Na_2O**	0.94	0.77	0.55	0.69-0.77	0.74	0.72	0.70	0.69	0.73	0.77	0.70	0.69	0.70	0.73
**MnO**	0.95	0.83	0.03	0.75-0.81	0.80	0.79	0.77	0.77	0.76	0.78	0.81	0.75	0.79	0.80
**TiO_2**	0.96	0.80	0.16	0.73-0.77	0.74	0.74	0.77	0.76	0.75	0.75	0.73	0.74	0.77	0.77
**P_2O_5**	0.95	0.81	0.06	0.72-0.77	0.75	0.74	0.72	0.76	0.76	0.77	0.79	0.76	0.75	0.77
**La**	0.95	0.77	12.33	0.69-0.77	0.69	0.69	0.70	0.73	0.74	0.77	0.73	0.75	0.73	0.69
**Ce**	0.95	0.76	23.65	0.67-0.77	0.71	0.67	0.77	0.71	0.71	0.70	0.73	0.71	0.72	0.71
**Nd**	0.94	0.75	10.05	0.64-0.75	0.69	0.69	0.64	0.67	0.70	0.69	0.73	0.72	0.67	0.71
**Sm**	0.95	0.77	1.86	0.69-0.79	0.72	0.77	0.74	0.70	0.79	0.67	0.70	0.70	0.69	0.76
**Eu**	0.94	0.78	0.34	0.71-0.78	0.76	0.73	0.75	0.74	0.78	0.73	0.71	0.72	0.72	0.73
**Gd**	0.94	0.77	1.69	0.68-0.78	0.70	0.73	0.68	0.68	0.74	0.74	0.73	0.78	0.69	0.74
**Dy**	0.95	0.79	1.57	0.70-0.80	0.75	0.75	0.70	0.73	0.68	0.79	0.80	0.73	0.75	0.76
**Yb**	0.95	0.80	0.98	0.68-0.79	0.75	0.70	0.68	0.68	0.75	0.69	0.73	0.70	0.79	0.71
**Sr**	0.92	0.80	152.17	0.74-0.80	0.76	0.76	0.78	0.75	0.79	0.76	0.74	0.79	0.75	0.79
**Y**	0.95	0.82	7.54	0.74-0.81	0.78	0.76	0.79	0.71	0.81	0.77	0.74	0.79	0.75	0.74
**Nb**	0.93	0.83	9.18	0.71-0.84	0.84	0.80	0.79	0.84	0.83	0.82	0.83	0.71	0.74	0.83
**Th**	0.91	0.80	5.14	0.73-0.78	0.75	0.78	0.77	0.76	0.72	0.75	0.75	0.75	0.73	0.73

References

1. Wang L, Liu J, Spencer CJ *et al.* The Role of Continental Alkaline Magmatism in Mantle Carbon Outflux Constrained by a Machine Learning Analysis of Zircon. *Geophys Res Lett* 2024;**51**:e2023GL106847.

2. Bell EA, Boehnke P, Harrison TM. Recovering the primary geochemistry of Jack Hills zircons through quantitative estimates of chemical alteration. *Geochim Cosmochim Acta* 2016;**191**:187–202.

3. Ge R-F, Wilde SA, Zhu W-B *et al.* Earth's early continental crust formed from wet and oxidizing arc magmas. *Nature* 2023;**623**:334–9.

4. Laurent O, Moyen J-F, Wotzlaw J-F *et al.* Early Earth zircons formed in residual granitic melts produced by tonalite differentiation. *Geology* 2022;**50**:437–41.

5. Sun G, Liu S, Cawood PA *et al.* Thermal state and evolving geodynamic regimes of the Meso- to Neoarchean North China Craton. *Nat Commun* 2021;**12**:3888.

6. Bergen KJ, Johnson PA, De Hoop MV *et al.* Machine learning for data-driven discovery in solid Earth geoscience. *Science* 2019;**363**:eaau0323.

7. Chen T, Guestrin C. XGBoost: A Scalable Tree Boosting System. *Proceedings of the 22nd ACM SIGKDD International Conference on Knowledge Discovery and Data Mining*. San Francisco California USA: ACM, 2016, 785–94.

8. Middlemost EAK. Naming materials in the magma/igneous rock system. *Earth-Sci Rev* 1994;**37**:215–24.

9. Rollinson HR. *Using Geochemical Data: Evaluation, Presentation, Interpretation*. London: Routledge, 2013.

10. Sun S -s., McDonough WF. Chemical and isotopic systematics of oceanic basalts: implications for mantle composition and processes. *Geol Soc Lond Spec Publ* 1989;**42**:313–45.

11. Reimink JR, Chacko T, Stern RA *et al.* Earth's earliest evolved crust generated in an Iceland-like setting. *Nat Geosci* 2014;**7**:529–33.

12. Lu T-Y, He Z-Y, Klemd R. Identifying crystal accumulation and melt extraction during formation of high-silica granite. *Geology* 2022;**50**:216–21.

13. Wang H, Yang J-H, Zhu Y-S *et al.* Archean crustal growth and reworking revealed by combined U-Pb-Hf-O isotope and trace element data of detrital zircons from ancient and modern river sediments of the eastern Kaapvaal Craton. *Geochim Cosmochim Acta* 2022;**320**:79–104.

14. O'Conner JT. A classification of quartz-rich igneous rocks based on feldspar ratios. *Us Geol Surv Prof Pap* 1965.

15. Moyen J-F. The composite Archaean grey gneisses: Petrological significance, and evidence for a non-unique tectonic setting for Archaean crustal growth. *Lithos* 2011;**123**:21–36.

16. Zhu D-C, Wang Q, Weinberg RF *et al.* Interplay between oceanic subduction and continental collision in building continental crust. *Nat Commun* 2022;**13**:7141.

17. Schiano P, Monzier M, Eissen J-P *et al.* Simple mixing as the major control of the evolution of volcanic suites in the Ecuadorian Andes. *Contrib Mineral Petrol* 2010;**160**:297–312.

18. Li Z-X, Liu Y, Ernst R. A dynamic 2000—540 Ma Earth history: From cratonic amalgamation to the age of supercontinent cycle. *Earth-Sci Rev* 2023;**238**:104336.

19. Cawood PA, Chowdhury P, Mulder JA *et al.* Secular Evolution of Continents and the Earth System. *Rev Geophys* 2022;**60**:e2022RG000789.
